# Tracking tumor heterogeneity and progression with near‐infrared II fluorophores

**DOI:** 10.1002/EXP.20220011

**Published:** 2023-03-16

**Authors:** Qi Xin, Huizhen Ma, Hao Wang, Xiao‐Dong Zhang

**Affiliations:** ^1^ Tianjin Key Laboratory of Brain Science and Neural Engineering Academy of Medical Engineering and Translational Medicine, Tianjin University Tianjin China; ^2^ Department of Pathology Tianjin Third Central Hospital, Tianjin Key Laboratory of Extracorporeal Life Support for Critical Diseases Tianjin China; ^3^ Department of Physics and Tianjin Key Laboratory of Low Dimensional Materials Physics and Preparing Technology, School of Sciences Tianjin University Tianjin China

**Keywords:** bioimaging, NIR‐II fluorophores, tumor heterogeneity, tumor progression

## Abstract

Heterogeneous cells are the main feature of tumors with unique genetic and phenotypic characteristics, which can stimulate differentially the progression, metastasis, and drug resistance. Importantly, heterogeneity is pervasive in human malignant tumors, and identification of the degree of tumor heterogeneity in individual tumors and progression is a critical task for tumor treatment. However, current medical tests cannot meet these needs; in particular, the need for noninvasive visualization of single‐cell heterogeneity. Near‐infrared II (NIR‐II, 1000–1700 nm) imaging exhibits an exciting prospect for non‐invasive monitoring due to the high temporal‐spatial resolution. More importantly, NIR‐II imaging displays more extended tissue penetration depths and reduced tissue backgrounds because of the significantly lower photon scattering and tissue autofluorescence than traditional the near‐infrared I (NIR‐I) imaging. In this review, we summarize systematically the advances made in NIR‐II in tumor imaging, especially in the detection of tumor heterogeneity and progression as well as in tumor treatment. As a non‐invasive visual inspection modality, NIR‐II imaging shows promising prospects for understanding the differences in tumor heterogeneity and progression and is envisioned to have the potential to be used clinically.

## Graphical abstract

The applications of NIR‐II Fluorophores in tracking the heterogeneity and progression of tumors. The advances in imaging the tumor heterogeneity, progression, and therapy are systematically summarized. As a non‐invasive visualized inspection modality, NIR‐II imaging further enables a deeper understanding of tumor pathophysiology and is envisioned for potential clinical translation.

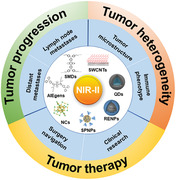

## 1 INTRODUCTION

As the most common cause of death worldwide, the morbidity of tumors presents an increasing trend,^[^ ^1^ ^]^ and the prognosis of tumors can be affected by several critical factors, including monitoring the progression of tumors, making early diagnoses, and the accurate surgical removal. However, the tumor progression with the complex process is not so easy to be monitored due to the tumor proliferation, invasion, adhesion, metastasis, and angiogenesis, as well as complex molecular and metabolic changes. Due to the evolution of tumors, tumor cells promote the transformation of phenotypically normal cells into malignant cells and promote the progression of malignant cells while sacrificing or utilizing host tissues. The following process begins after the establishment of malignancy: (1) local invasion via the extracellular matrix, (2) intravasation into the vascular lumina, (3) retention at distant organ sites through the vasculature, and (4) survival as a microtransaction in external microenvironments, leading to macroscopic tumor growth in clinical.^[^ ^2^ ^]^ Thus, it is crucial to develop a noninvasive method for monitoring tumor progression in real‐time.

Additionally, the heterogeneity of tumor cells resulting in chemoresistance, metastasis, and progression impedes the diagnosis and treatment and should be taken seriously.^[^ ^3^ ^]^ Tumor heterogeneity includes inter‐ and intra‐tumor heterogeneity. Inter‐tumor heterogeneity means the differences within individual tumors, which can be subdivided into spatial (different regions of the same tumor) and temporal heterogeneity (distinctions between the original tumor and metastatic tumor).^[^ ^4^, ^5^ ^]^ Furthermore, heterogeneity constantly changes as tumors progress.^[^ ^6^ ^]^ The evolutionary process of tumors is extremely complex due to the change in tumor heterogeneity at different stages.^[^ ^7^ ^]^ Additionally, phenotypic and functional heterogeneity will be present in tumor cells within the same tumor due to objective factors.^[^ ^8^ ^]^ The heterogeneity of the primary tumor has likely been transformed when solid tumors progressed further or recurred after systemic therapy for metastatic disease.^[^ ^9^ ^]^ Current detection of tumor heterogeneity is based on biopsy through immunohistochemical and genetic tests. Direct comparison of multiple metastatic samples from the same patient is the most rational to study the heterogeneity change in the progression of metastasis. However, obtaining individual biopsies from the same patient at various stages of the disease is challenging at the moment.^[^ ^3^, ^10^ ^]^ Therefore, a non‐invasive and non‐destructive imaging technique is urgently needed to evaluate tumor heterogeneity.

Determination of tumor origin and differentiation is an important basis for evaluating patient prognostic and therapeutic responses. Therefore, one of the essential tasks for tumor diagnosis is to identify the origin of tumor tissue. Two main approaches can be performed to mark tumors by using NIR‐II fluorescence. One is the nonspecific method by utilizing the enhanced permeability and retention (EPR) effect,^[^ ^11^ ^]^ another one is molecular imaging by employing specific targeting ligands.^[^ ^12^, ^13^ ^]^ The majority of cancer biomarkers have been identified as perfect targets for the diagnosis of cancer, such as epidermal growth factor receptor (EGFR), transferrin receptor, human epidermal growth factor receptor 2 (HER2), folate receptor, endothelin receptor, integrin receptor, and prostate specific membrane antigen (PSMA).^[^ ^14^ ^]^

**FIGURE 1:**
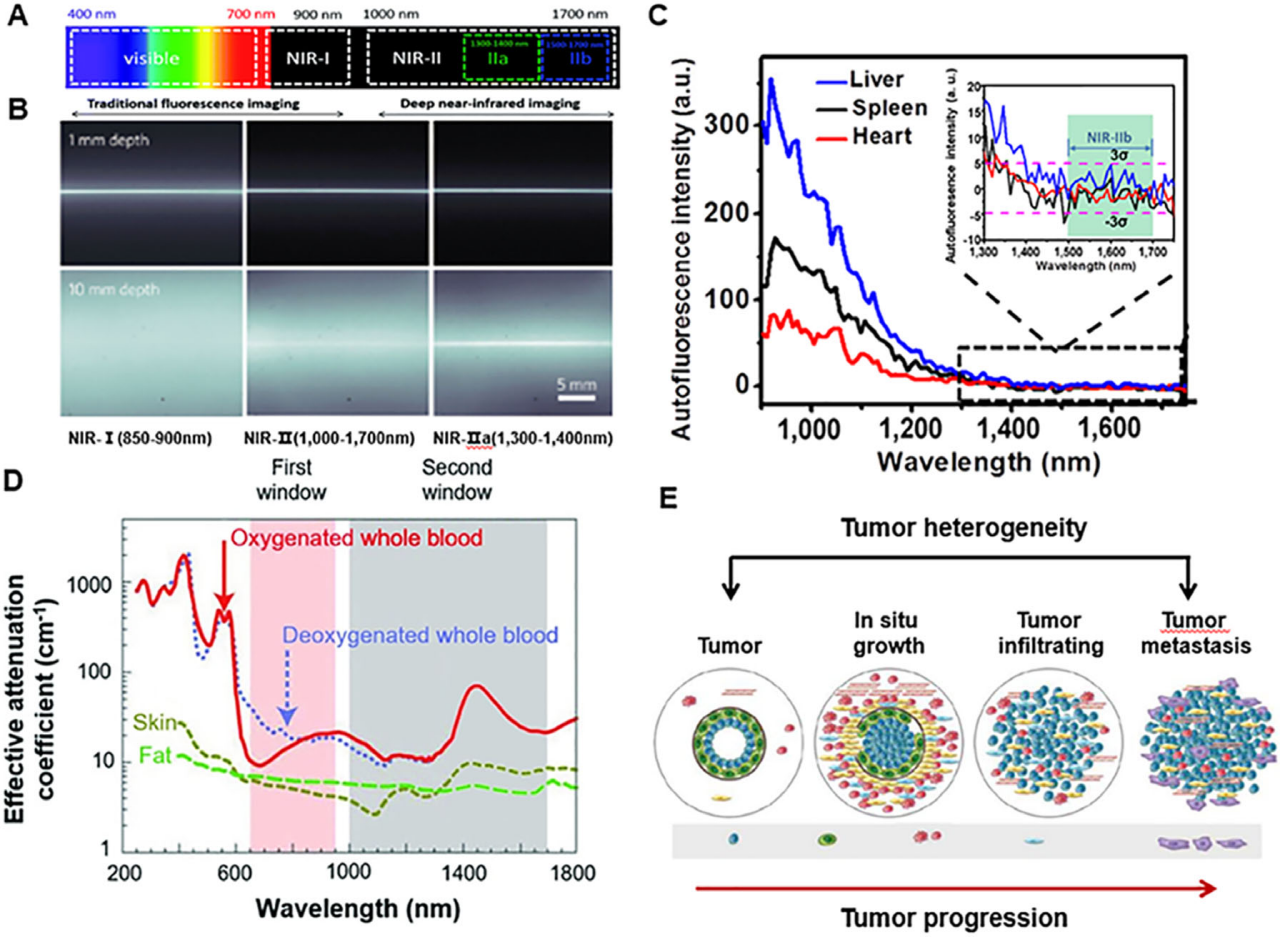
NIR‐II window for tumor imaging. (A) Segmentation of wavelengths of light. Reproduced with permission.^[^ ^312^ ^]^ Copyright 2018, Royal Society of Chemistry. (B) Depths of penetration of the light at different wavelengths. Reproduced with permission.^[^ ^24^ ^]^ Copyright 2014, Springer Nature. (C) Autofluorescence of ex vivo mouse liver (blue), spleen (black), and heart tissue (red) disappears at >1500 nm wavelengths. Reproduced with permission.^[^ ^27^ ^]^ Copyright 2015, Springer Nature. (D) Effective attenuation coefficients of skin, fat, and blood. Reproduced with permission.^[^ ^27^ ^]^ Copyright 2009, Springer Nature. (E) The heterogeneity and progression of the tumor.

Biomedical imaging plays an indispensable role in fundamental research and clinical applications.^[^ ^15^, ^16^ ^]^ Traditional imaging techniques do not meet the current requirements for inspecting tumor heterogeneity and progression due to intrinsic shortcomings. High resolution magnetic resonance imaging (MRI) systems with high costs exhibit slow dynamic imaging.^[^ ^17^ ^]^ Ultrasound (US) imaging does not provide structural and functional information at high resolution.^[^ ^18^ ^]^ Computed tomography (CT) and positron emission tomography (PET) are not appropriate for frequent imaging due to ionizing radiation.^[^ ^19^ ^]^ Fluorescence imaging shows unique advantages, such as high spatiotemporal resolution, excellent sensitivity, flexible selectivity, superior visualization, real‐time analysis, and positioning of biological molecules.^[^ ^20^, ^21^, ^22^, ^23^ ^]^ As the wavelength of fluorescence increases, the penetration depth of light can be increased (Figure 1A,B),^[^ ^24^ ^]^ and the tissue absorption and interference with autofluorescence can also be diminished.^[^ ^25^ ^]^

NIR‐I (700–900 nm) exhibited reduced scattering and absorption compared to visible light (400–700 nm) fluorescence imaging. NIR‐I can penetrate biological components more deeply and effectively, especially the skin and blood, achieving a tissue penetration depth of approximately 1 mm. NIR‐I fluorophores are mainly used as a beneficial tool for biomedical imaging at the NIR‐I window.^[^ ^26^ ^]^ Correspondingly, some materials with NIR‐I fluorescence have been extensively used in preclinical research and clinical medicine. For example, indocyanine green (ICG) and methylene blue (MB) have been developed to be applied in clinical practice. However, the NIR‐II (1000–1700 nm) imaging, also known as shortwave infrared imaging (SWIR, 1000–1700 nm), provides superior fluorescence image quality compared to that of NIR‐I. The NIR‐II window has deep tissue penetration with centimeter‐scale due to reduced scattering, ignorable tissue absorption, and ultra‐low autofluorescence, which achieves higher resolution with micrometer‐scale. In particular, the autofluorescence of living tissues disappears at wavelengths greater than 1500 nm (Figure 1C,D).^[^ ^25^, ^27^ ^]^ With the simple bioconjugation with peptides/proteins of specific functions, NIR‐II fluorophores are exploited as great promising candidates for tumor‐targeted imaging. Currently, small size and low molecular weight moieties with binding specificities and affinities for targets similar to commonly used antibodies have been exploited. For example, microRNA‐21, a model target that binds to the surface of graphene oxide by π‐stacking interactions between graphene oxide and nucleobases, has been used to design a graphene oxide probe encapsulated with nanocrystals for intracellular tracking and cancer biomarker imaging.^[^ ^28^ ^]^

Based on the above advantages, NIR‐II imaging is more suitable for tumor imaging, especially for monitoring tumor progression in real‐time. Tumor imaging has attracted extensive attention over the past decade (Figure 1E). The research efforts mainly focused on early‐stage tumor‐targeted imaging, fluorescence‐guided surgery, tumor progression, and therapy.^[^ ^29^, ^30^, ^31^, ^32^, ^33^ ^]^ In recent years, great advances were made in NIR‐II fluorophore design and biological application. The organic dyes for cancer imaging and surgery with NIR‐II fluorescence are reviewed by Zhu'sgroup.^[^ ^34^ ^]^ The synthesis method, chemical structure, physicochemical properties, bioconjugation, and biological behavior were summarized. Meanwhile, bioimaging for cancer detection, vessel imaging, and lymphatic imaging is also been displayed in their work. The latest developments of NIR‐II fluorophores and their applications in biomedicine were reviewed by Chen et al. The NIR‐II fluorophores can be rationally designed for use in multiple fields, especially in tumor removal, image navigation, blood vessel imaging, quantitative drug release, photothermal therapy (PTT), and photodynamic therapy (PDT).^[^ ^35^ ^]^ In this review, the current development of various NIR‐II fluorophores and their application in tumor heterogeneity and tumor progression are summarized. In particular, a comprehensive overview of the progression and treatment of tumor heterogeneity is presented. More importantly, we discuss the potential prognostic value of NIR‐II probes for detecting these changes in heterogeneity during tumor progression.

## 2 NIR‐II FLUOROPHORES

**FIGURE 2:**
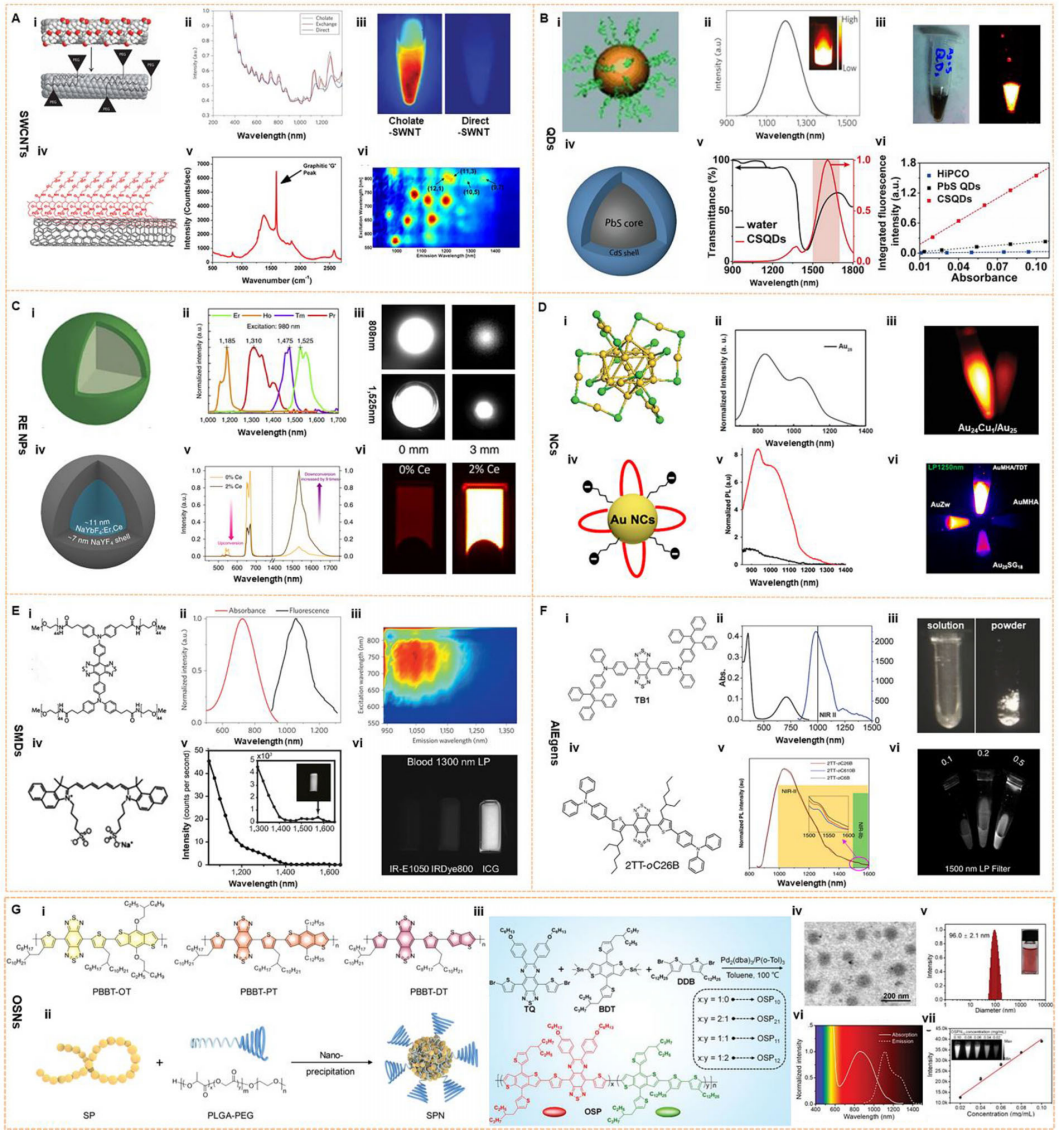
Fluorophores dyes for tumor imaging on NIR‐II window. (A) The first SWNT for NIR‐II imaging; Reproduced with permission.^[^ ^43^ ^]^ Copyright 2009, Springer Nature. Characterization of C_18_‐PMH‐mPEG coated SWNTs. Reproduced with permission.^[^ ^40^ ^]^ Copyright 2012, American Chemical Society. (B) A scheme shows the 6 PEG‐Ag_2_S QDs that emit at 1200 nm upon excitation at 808 nm; Reproduced with permission.^[^ ^63^ ^]^ Copyright 2012, John Wiley & Sons. The fluorescence emission spectrum of CSQDs, and plot of the integrated fluorescence spectra of PEG‐CSQDs at five different absorbance values of 808 nm. Reproduced with permission.^[^ ^45^ ^]^ Copyright 2018, National Academy of Sciences. (C) NaYF_4_ Yb: Core–shell structure of Ln (REs) and its NIR‐II emission spectra. Reproduced with permission.^[^ ^46^ ^]^ Copyright 2013, Springer Nature. Ce^3+^‐doped Er‐RE NPs with enhanced NIR‐IIb luminescence. Er‐RE NPs with 2% Ce doping have high QY. Reproduced with permission.^[^ ^74^ ^]^ Copyright 2017, Springer Nature. (D) An atomic‐precision gold cluster with 25 gold atoms and 18 peptide ligands (Au_25_(SG)_18_). Reproduced with permission.^[^ ^55^ ^]^ Copyright 2019, John Wiley & Sons. Scheme of the Au NCs AuMHA/TDT, PL spectra of AuMHA (black line) and AuMHA/TDT (red line) (λ_exc._ 830 nm), and the fluorescence intensity of AuMHA and AuMHA/TDT under NIR excitation_._ 830 nm using long‐pass (LP) 1250 nm (5 ms). Reproduced with permission.^[^ ^88^ ^]^ Copyright 2020, American Chemical Society. (E) The first generation of D‐A‐D type small‐molecule NIR‐II organic fluorophore: CH1055. Reproduced with permission.^[^ ^106^ ^]^ Copyright 2015, Springer Nature. Structure and emission peak of ICG on the NIR‐II window. The right side: the NIR‐II fluorescence intensity of ICG (0.01 mg/mL) in bovine blood with a 1300 nm LP filter. Reproduced with permission.^[^ ^119^ ^]^ Copyright 2018, National Academy of Sciences. (F) Chemical structure, absorption, and emission spectra of TB1; NIR‐II fluorescence images of a DMSO solution (left) with 10 ms exposure time and solid powder (right) under an excitation of 808 nm at 45 mW cm^−2^, respectively. Reproduced with permission.^[^ ^56^ ^]^ Copyright 2018, John Wiley & Sons. Chemical structure and emission spectra of 2TT‐oC26B, NIR‐II signals at different concentrations (mg/mL) (right). Reproduced with permission.^[^ ^101^ ^]^ Copyright 2020, Springer Nature. (G) Chemical structures and preparation method of NIR‐II PA OSNs. Reproduced with permission.^[^ ^49^ ^]^ Copyright 2019, John Wiley & Sons. Synthetic route of OSNs with various doping ratios and chemical and physical properties of OSNs. Reproduced with permission.^[^ ^139^ ^]^ Copyright 2021, John Wiley & Sons.

The design of NIR‐II fluorophores with excellent performance is a crucial technology for biomedical monitoring and functional imaging.^[^ ^29^, ^36^, ^37^ ^]^ In recent decades, the development of imaging has driven rapid progress in the field of materials.^[^ ^38^, ^39^ ^]^ As shown in Figure 2, researchers have successively developed various fluorophores for tumor imaging with NIR‐II fluorescence, such as single‐walled carbon nanotubes (SWCNTs),^[^ ^40^, ^41^, ^42^, ^43^ ^]^ quantum dots (QDs),^[^ ^44^, ^45^ ^]^ rare‐earth nanoparticles (RE NPs),^[^ ^46^, ^47^, ^48^ ^]^ organic semiconducting polymers nanoparticles (OSNs),^[^ ^49^, ^50^ ^]^ small molecular dyes (SMDs),^[^ ^51^, ^52^, ^53^, ^54^ ^]^ nanoclusters (NCs),^[^ ^55^ ^]^ and aggregation‐induced luminescence groups (AIEgens).^[^ ^56^, ^57^, ^58^, ^59^ ^]^ The development of NIR‐II dyes expands the range of fluorophores for tumor imaging applications (Table 1).

**TABLE 1:** Summary of the properties and applications of representative NIR‐II.

Name	Ex (nm)	Em (nm)	Application	Properties	QY (%)	Biocompatibility	Ref.
SWNT	785/808	1000–1700	Deep‐tissue anatomical imaging Real‐time assessment of a blood flow anomaly in middle cerebral artery occlusion stroke model Brain imaging Imaging of mouse hind limb vasculatures structures Differentiation of arterial and venous vessels Calculation of femoral artery blood flow Tumor and the vessels imaging Intravital microscopy imaging of tumor vessels Image‐guided surgery	Be coated with C18‐PMH‐mPEG Exchange‐SWNTs Conjugated with IRDye‐800 Conjugated with M13	0.4–0.84	Water‐soluble In dimethylsulphoxide(DMSO) With 75% DSPE‐mPEG	[24, 40, 41, 42, 43, 184]
SA@SWCNT	808	1500–1700	Tumor vessel sections imaging	Conjugated with CD31‐biotin	NA	With PEG chains	[61]
CS QD	808	1600	Tumor imaging Tumor microvasculature imaging Hind limb vasculatures imaging Tumor immunity imaging	Conjugated with PD‐1	2.2–22	Aqueous solutions	[45, 208]
Ag_2_S QD	808	1200	Detection of the tumor Pinpointing of the location of the tumor Lymphatic system imaging Blood pool imaging Angiogenesis of tiny tumor imaging Image‐guided surgery Peritoneal metastasis tumor detection	Conjugated with RGD	15.5	With blood‐circulation half‐life of 37 ± 0.75 h	[63, 189, 257]
SWIR QD	808	900–1600	Metabolic imaging High‐speed whole‐body imaging Intravital the vessel structure of glioblastoma imaging		30		[44]
PEG‐PATU Ag_2_S QD	785	1110	Tumor cell tracking In vivo real‐time imaging of the vascular system		7.8	Excellent water‐dispersibility Fast cellular uptake	[64]
CSS	532	1296	Cerebrovascular imaging		82	Water‐soluble	[171]
Ag_2_S superdots	808	1200	In vivo vessel imaging		10.7	A low in vivo toxicity	[66]
PbS@CdS QDs	785	1200	Gastrointestinal tract imaging		61	Low toxicity	[69]
NaYF4	980	1550–1600	Tumor detection	Albumin‐coated	4.2	Great cell viability	[46]
CSS	800	1000–1600	Deep optical bioimaging		4.2		[48]
Er‐RENP	980	1550	Cerebrovascular imaging		0.27–2.73	Conjugation of (mPEG‐NH_2_)	[74]
Nd@PEG	808	1060	Tumor targeted imaging Image‐guided surgery	Cancer cell membrane coated	7.1	Low toxicity	[75]
Core‐multi‐shell nanoparticle	808	1155/ 1525	Breast cancer diagnostics	Conjugated with ER, PR, and HER2	NA	Aqueous solubility	[76]
PAA‐C/S	808	1525	Lung tumor vascular imaging Colorectal tumor vascular imaging Delineation of the tumor margin Image‐guided surgery		2.9	High cell viability	[160]
DCNP	808	1060	Tumor metastasis imaging Distinction of the tumor margin Image‐guided surgery	Conjugated with FSH_β_	NA		[180, 288]
α‐ErNP	980	1550	In vivo PD‐L1 molecular imaging	Conjugated with atezolizumab	5	Low toxicity	[207]
Au_25_ cluster	808	1120	Lymph nodes imaging Tumor metastasis imaging In vivo brain vessel imaging Monitor high resolution imaging of kidney		0.67	Low toxicity	[55]
AuMHA/TDT	830	1250–1700	Detect vascular disorders		6	Water‐soluble	[88]
CD‐Au NCs	808	1050	Tumor targeted imaging	Conjugated with CD326	0.11		[96]
RNase‐A@AuNC	808	1050	Diagnosis intestinal tumor	RNase‐A‐encapsulated	1.9	With eligible toxicity	[223]
CH1055	750	1055	In vivo brain vessel imaging Lymphatic imaging Noninvasive imaging of brain tumor Image‐guided surgery	Conjugated with anti‐EGFR	0.3		[24, 106]
IR‐26	1080	1130	NA		0.5		[105, 118]
IR‐1061	808	1132	Hindlimb vessel imaging Abdomen of nude mice imaging		1.8		[128]
IR‐FGP	808	900–1400	3D layer‐by‐layer molecular imaging of SCC	Conjugated with EGFR	1.9		[61]
IR‐FTAP	733	1048	Ultrafast vessel imaging		5.3		[112]
CP‐IRT	808	1050	Subcutaneous human colon cancer image	Conjugated with CD133	1.5	Low toxicity	[165]
IR‐BEMC6P	725	1025	Orthotopic brain tumor imaging The brain vessels imaging The AR42J tumor imaging Distinction of the tumor margin Image‐guided micro‐surgery	Conjugated with RGD peptide Conjugated with the octreotide peptide Conjugated with FSH	1.8		[166]
m‐PBTQ4F Pdots	808	1300	Whole mouse blood vessels imaging Cerebral vasculature imaging Reveal the vascular characteristics of medulloblastoma		3.2		[172]
TQFP‐10	808	1021	Subcutaneous glioblastoma imaging Orthotopic glioblastoma imaging		7.6	Low toxicity	[178]
Q4	808	1000	Vessels of the tumor imaging Targeted imaging of prostate cancer	Conjugated with RM26	0.2		[107]
p‐FE	808	1010	Cerebral vasculature imaging Hindlimb vasculatures Tumor structure imaging		16.5		[191]
IR‐FD	800	1100	Lymph node‐invaded cancer detection Imaging‐guided surgery		6.0		[242]
IRBTP‐B	808	900–1500	Drug‐induced hepatotoxicity monitoring		0.05		[52]
CQS1000	830	1000–1200	Hind limb and cerebrovascular imaging Assessment of angiogenesis of tumor Image‐guided surgery Imaging for arterial thrombus formation and incomplete hind limb ischemia Lymphatic drainage imaging Imaging‐guided sentinel lymph node mapping and biopsy				[16]
H1	785	1100	Imaging of blood vessels on tumors Image‐guided sentinel lymph node surgery		2.0	Low cell toxicity	[108]
FM1210	808	1210	Abdominal vascular imaging Imaging a tumor and its vasculature		0.036		[53]
PDFT1032	808	1000–1200	Subcutaneous osteosarcoma imaging Image‐guided surgery Micrometastasis detection The vascular embolization therapy of osteosarcoma Image‐guided sentinel lymph node biopsy				[162]
P1	808	1064	Orthotopic brain tumor imaging Image‐guided surgery	Conjugated with RGD	2	Low toxicity	[168]
Flav7	808	1000–1600	Vasculature of the hind limb imaging		0.53	High cell viability	[122]
5H5	1069	1125	Abdominal vessels imaging Tumor microvasculature imaging Nonspecific tumor imaging αVβ3‐targeted U87MG tumor imaging	Conjugated with RGD	2.6		[123]
CX‐3	1089	1140	Lymph node imaging Drug‐induced hepatotoxicity monitoring		0.091		[124]
FD‐1080	1064	1080	Hindlimb vasculature imaging In vivo cerebrovascular imaging		0.31		[125]
FD‐1080 J‐aggregates	1360	1370	Hindlimb vasculature imaging In vivo cerebrovascular imaging Dynamic imaging of the carotid artery width Monitor blood pressure		0.0545		[127]
PCP‐BDP2 J‐aggregates	750	1010	Hindlimb vasculature imaging In vivo cerebrovascular imaging Lymph node imaging Image‐guided surgery		6.4		[245]
FD‐1029	977	1029	Abdominal vascular imaging Lymphatic imaging Tumor imaging Tumor metastases tracing		0.029	High cell survival rate	[252]
ICG	780	830	Human liver‐tumor surgery guided Visualization of the biliary tract structures		9.3		[25, 119, 293]
IRDye800CW	774	789	Image‐guided surgery		12	No toxic reactions	[114]
HL3	808	1550	Whole mouse blood vessels imaging Cerebral vasculature imaging Lymph node imaging		0.05	High cell viability	[54]
2TT‐oC26B	730	1031	Whole‐body imaging of living mice Cerebral vasculature imaging Bowel imaging		11.5		[101]
2TT‐oC6B	733	1030	Intraoperative identification of ureter		11	Low toxicity	[57]
TB1	740	1000	Orthotopic brain tumor imaging Cerebral vasculature imaging Brain tumor structure imaging	Conjugated with c‐RGD	6.2		[56]
pNIR‐4	750	1040	Cerebral vasculature imaging Hindlimb vasculature imaging Image‐guided surgery Lymphatic imaging		2.24		[58]
SA‐TTB‐PEG 1000	728	1050	Whole‐body vasculature imaging of living mice Hindlimb vasculature imaging The vasculature in mouse ear imaging		10.3		[190]
BTPETQ	700	1200	Cerebral vasculature imaging The differentiation of normal blood vessels from tumor vessels		19	High cell viability	[192]
BPBBT	831	1100	Identified primary orthotopic colon Identified metastatic orthotopic colon Image‐guided surgery		1.45		[259]

Abbreviations: Ex: excitation wavelength; Em: emission wavelength; QY: quantum yield; NA: not applicable.

### 2.1 SWCNTs

**FIGURE 3:**
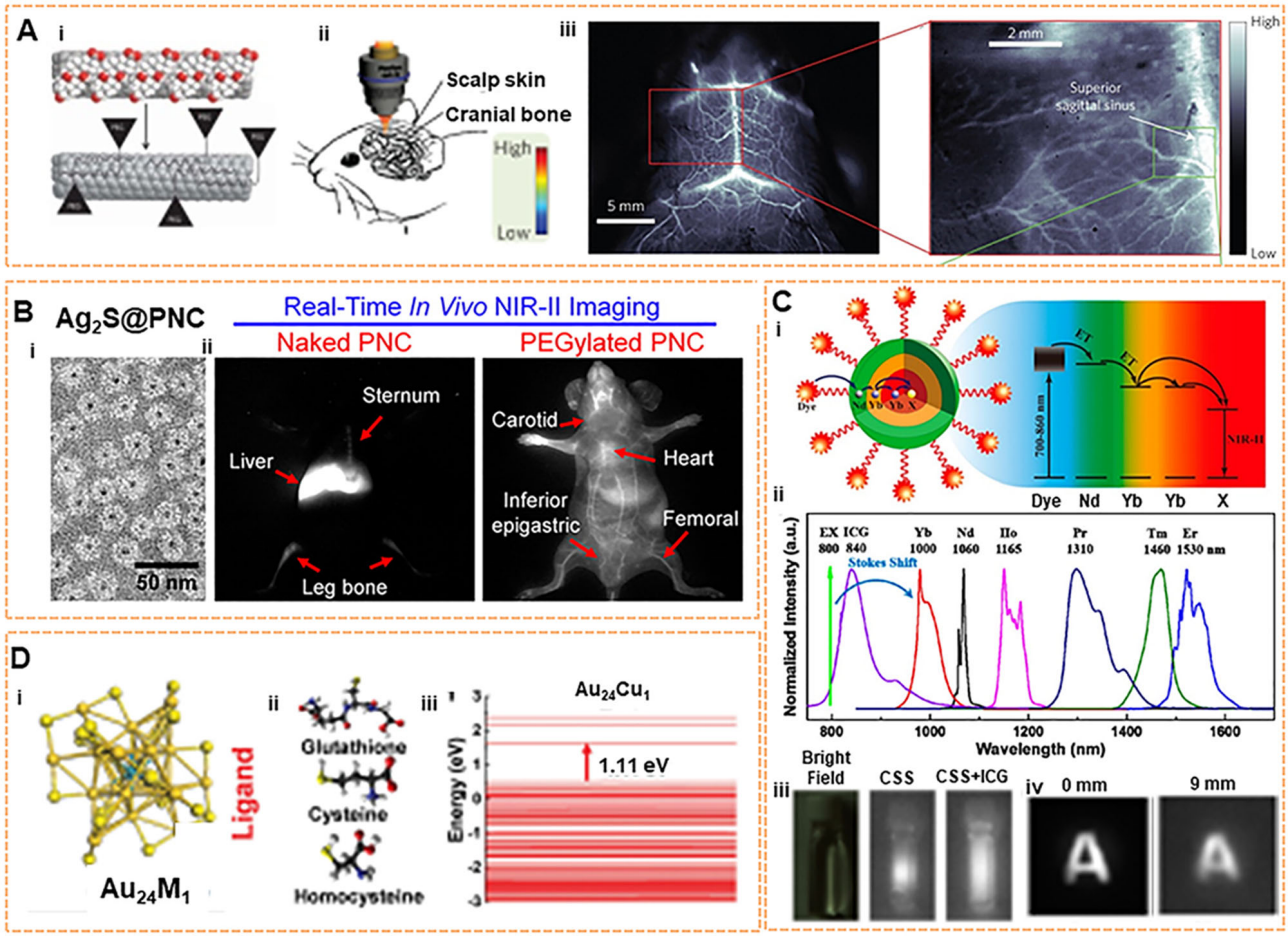
Inorganic fluorophores for NIR‐II imaging. (A) SWCNT for cerebrovascular imaging in mice. Reproduced with permission.^[^ ^24^ ^]^ Copyright 2014, Springer Nature. (B) The real‐time monitoring with Ag_2_S QD at the NIR‐II window and the chemical‐dependent surface behavior of protein nanocages in mice. Reproduced with permission.^[^ ^65^ ^]^ Copyright 2015, American Chemical Society. (C) The energy transfer pathway of ICG‐sensitized CSS nanocrystals, NIR‐II emission spectra, and NIR‐II photoluminescent image. Reproduced with permission.^[^ ^48^ ^]^ Copyright 2016, American Chemical Society. (D) Crystal structure of Au_24_M_1_, the schematic of three ligands, and HOMO‐LUMO band gap of Au_24_M_1_ cluster. Reproduced with permission.^[^ ^55^ ^]^ Copyright 2019, John Wiley & Sons.

Inorganic nanomaterials exhibit excellent quantum yield (QY) and enormous photobleaching resistance, promoting them as a promising tool for NIR‐II fluorescence imaging. In 2009, Dai described the first application of SWCNTs with NIR‐II luminescence and achieved the first high‐resolution imaging with NIR‐II in living mice using self‐built imaging equipment (Figure 3A).^[^ ^43^ ^]^ The SWCNTs with low toxicity and reticuloendothelial system excretion could obtain high brightness and excellent biocompatible by surfactant exchange. In 2012, the SWNTs could be solubilized by using the novel synthetic polymer, realizing the tumor imaging at the NIR‐II window.^[^ ^40^ ^]^ Nevertheless, the water‐soluble SWNTs with DSPE‐mPEG (PEG: polyethylene glycol) still have residues in the spleen. In 2014, SWCNTs were exploited to achieve noninvasive imaging of the mouse brain with NIR‐IIa fluorescence,^[^ ^60^ ^]^ achieving high resolution imaging with a depth of over 2 mm and a high resolution. The remarkable progress of SWNTs has been performed for tumor fluorescence imaging over the past few years, demonstrating the substantial potential in tumor research. Robinson et al. synthesized a high‐performance SWNT with a circulating half‐life of 30 h in vivo.^[^ ^40^ ^]^ In addition, high resolution in vivo microscopy imaging was also achieved, achieving visualization of small tumor vessels.^[^ ^25^ ^]^ Moreover, the colocalization of the tumor vasculatures and SWNTs can be demonstrated by the 3D reconstruction of NIR‐II fluorescence.^[^ ^61^ ^]^ A clickable organic fluorophore and a carbon nanotube fluorescent agent have been designed by conjugating molecularly specific proteins or antibodies to perform targeted molecular imaging of tumors.^[^ ^61^ ^]^ However, due to the low fluorescence QY of SWCNTs (< 1%) and poor biocompatibility, their application is limited in biological imaging.

### 2.2 QDs

As well‐known NIR fluorophores, metal sulfide QDs have excellent optical properties due to narrow bandgap, intense brightness, relatively large absorption coefficient, and easy surface modification. The composition and size of QDs can be modified to adjust the optical properties and obtain new NIR‐II materials.^[^ ^62^ ^]^ However, the development of QDs is hindered due to biosafety. It is well known that the strong toxicity of QDs with heavy metal is mainly caused by the leaching of free heavy metal ions from nanoparticles. To solve this problem, researchers have explored biocompatible coatings on the surface of QDs, which reduce the release of toxic ions and enhance the fluorescence intensity by smoothing the nanoparticle surface. To date, QDs with NIR‐II luminescence have been reported, including Ag_2_S, Ag_2_Se, CdSe, PbS, and InAs. Among the plentiful available semiconductor QDs, Ag_2_S QDs are widely used in NIR‐II imaging for biological imaging due to the smaller particle size.^[^ ^63^, ^64^ ^]^ More significantly, their biotoxicity is lower than that of other QDs with other acute or chronic toxic elements. Wang QB team has performed considerable research on Ag_2_S QDs and developed the imaging technology platforms for monitoring in vivo based on Ag_2_S QDs. Furthermore, a series of biomedical “visualization” studies have been developed successively at the NIR‐II fluorescence window.^[^ ^38^, ^65^ ^]^ In 2015, a specific protein nanocage (PNC) encapsulated by Ag_2_S QD was proposed by the group to observe PNCs behavior in vivo via using the monkey virus PNC_SV40_ model.^[^ ^65^ ^]^ The dynamic distribution of PNC_SV40_ in vivo could be tracked with high fidelity due to the superiorities of deep‐tissue penetration, and high resolution of NIR‐II fluorescence. Furthermore, the surface chemical‐dependent behavior of PNC_SV40_ was revealed by adopting the PEG strategy (Figure 3B). However, most Ag_2_S@PNC_SV40_ and PEG Ag_2_S@PNC_SV40_ accumulate nonspecifically in the spleen and liver, causing potential toxicity in the long term. Regarding tumor NIR‐II imaging, Ag_2_S QDs coated with six‐armed PEG could be visualized in xenograft tumors through the EPR effect.^[^ ^63^ ^]^ For toxicity issues, the results illustrated that 6PEG‐Ag_2_S QDs are excreted at a stable rate mainly due to the larger size of 6PEG Ag_2_S QDs than renal filtration cut‐off size. Recently, the QY of Ag_2_S superdots (a new generation of Ag_2_S probes) could be increased from 0.13% to 10.7%, with an 80‐fold enhancement under 50‐min‐long irradiation by using 50 fs pulses.^[^ ^66^ ^]^ Besides Ag_2_S QDs, PbS QDs also exhibit great promise due to high QY and long emission wavelength (QY: 10%, *λ*_em_ = 1500–1700 nm).^[^ ^45^ ^]^ Nonetheless, biological applications are severely hindered due to the high toxicity of lead. The biocompatibility of PbS QDs can be increased by utilizing glutathione and protein as coatings.^[^ ^67^ ^]^ The emission wavelengths of PbS@CdS core–shell QDs can be expectedly regulated widely by adjusting the core diameter, achieving a high QY of 17%.^[^ ^68^ ^]^ Furthermore, the surface of PbS@CdS can be modified by using SiO_2_ and amphiphilic polymer (pluronic F‐127) coating, which benefits the dual‐layer protection of the PbS@CdS core and improves the stability whether in a highly acidic or alkaline environment.^[^ ^69^ ^]^ More importantly, no significant inflammation was found by using histopathological examination, proving that there was no obvious toxicity problem. As is well known to all, QDs have been widely recognized as excellent candidates for fluorescent dyes. To address these challenges mentioned above, it is necessary to design the desired fluorophore with higher QY and longer emission wavelengths. Hopefully, the QDs and QD‐based derivatives with superior optical properties will be achieved quickly based on the wide application of QDs in the field of biological imaging.

### 2.3 RE NPs

Composed of lanthanides, RE NPs with high stability and flexible wavelength tunability also play an important role as NIR‐II fluorophores, showing good prospects for biological imaging applications.^[^ ^46^, ^47^, ^48^ ^]^ Many RE NPs with core–shell structures have been developed based on the basic structure of RE nanocrystals. The rich energy levels with the electron configuration of 4f^n^5s^2^5p^6^ (*n* = 0–14) prompt the shift from ultraviolet (UV), visible to NIR. Essentially, The complex optical properties can be explained that the inner 4f–4f orbital electronic transitions filled by 5s and 5p orbitals of lanthanide ions are not sensitive to the outer environment.^[^ ^70^, ^71^ ^]^ Thus, RE NPs are endowed with sharp and tunable emissions with high photostability properties. RE NPs typically consist of a suitable inorganic host matrix, sensitizers, and activators. Among them, sensitizers and activators are generally composed of lanthanide ions of +3. Under laser irradiation with a specific wavelength, the electrons of the sensitizer are excited to the excited state from the ground state, and then the energy is transferred to the activator. The electrons return to the ground state which induces the excited activator to emit luminescence.^[^ ^72^ ^]^ Therefore, the luminescence mechanism of RE NPs can be greatly affected by the chemical composition, such as doping ions and matrix.

In 2018, the Chen team discovered the core–shell system of NaYF_4_ Yb: Ln. The fluorescence can be adjusted by controlling the ratio of lanthanide elements (such as Er, Ho, Tm, Pr) to obtain intense luminescence and a long fluorescence lifetime under 800 nm excitation.^[^ ^73^ ^]^ Experiments have shown that HeLa cell activity has not been significantly affected. In addition, in 2016, the Prasad team designed an organic‐inorganic hybrid system with the NaYF_4_: Yb^3+^/X^3+^@NaYbF_4_@NaYF_4_:Nd^3+^ (X = null, Er, Ho, Tm or, Pr) core‐shell–shell (CSS) nanocrystalline structure, which was sensitized by the organic molecule ICG (Figure 3C).^[^ ^48^ ^]^ A broad excitation band and multicolor narrow‐band NIR‐II emission peak of 1000–1600 nm could be provided by introducing the ICG. Depending on the different doping elements in the core, the intense brightness of approximately four‐fold from ICG‐sensitized CSS nanocrystals can be observed after sensitization. At a depth of 9 mm, the NIR‐II signal can still be captured by using ICG‐sensitized Er^3+^–doped CSS nanocrystals. Moreover, they have low biological toxicity by testing the HeLa cell viability. The emission of down‐conversion nanoparticle (DCNP) NaYF_4_ co‐doped with Yb/Er was demonstrated for melanoma imaging in vivo.^[^ ^46^ ^]^ The biocompatibility of the RE NPs has been evaluated in healthy, normal human epidermal melanocytes, revealing the low toxicity. Another Er‐based RE NP (NaYbF_4_:2%Er,2%Ce@NaYF_4_) with high QY was reported and the emission wavelength can be prolonged to 1550 nm under 980 nm excitation for NIR‐IIb imaging.^[^ ^74^ ^]^ Furthermore, cancer cell membrane‐coated RE NPs were used for tumor surgery navigation.^[^ ^75^ ^]^ RE NPs engineered with long luminescence lifetimes were used for time gating.^[^ ^73^ ^]^ A core/multishell structure (NaGdF_4_@NaGdF_4_: Yb/Er@NaYF_4_: Yb@NaNdF_4_: Yb) was also used for breast tumor diagnostics in vivo.^[^ ^76^ ^]^ The NaDyF_4_: Nd nanoprobe modified by a complex can precisely locate the tumor location and achieve tumor therapeutic efficacy.^[^ ^77^ ^]^ A large number of toxicity tests have been performed, such as MTT assay, fluorescence method using singlet oxygen sensor green (SOSG) and hematoxylin‐eosin (H&E) stained tissues sections, etc., which demonstrated the low toxicity of the RE NPs. Simultaneously, by comparing different LbL NIR‐II probes, rare‐earth‐based downconversion nanoparticles were regarded as diagnostic agents for high‐grade serous ovarian cancer and possessed the highest resolution of all tested probes.^[^ ^78^ ^]^ In 2022, Yang's group reported that the optimized ligand type gold nanoclusters (Au NCs) facilitated the rational construction of core‐satellite Ln@Au NCs components by using Er^3+^–doped lanthanide (Ln) nanoparticles, which enhanced the photoluminescence (PL) at 1100 nm and prolonged blood circulation.^[^ ^79^ ^]^ Therefore, the superior optical properties can be manipulated to achieve the desired results by utilizing lanthanides.^[^ ^80^ ^]^

### 2.4 NCs

It has recently been found that some metal clusters with excellent biocompatibility, intense brightness, and perfect photostability also possess NIR‐II fluorescence, such as Au or Ag clusters.^[^ ^81^, ^82^ ^]^ Ultrasmall NCs have demonstrated high levels of tumor accumulation due to their EPR effect.^[^ ^83^ ^]^ Moreover, cell activity experiments have been studied, proving the nontoxicity problems of NCs. Therefore, NCs have attracted more and more attention as novel imaging dyes for the NIR‐II region. NCs with relatively stable structures are multicore aggregates that contain several to several hundred atoms. Moreover, NCs with quantized charging and luminescence are endowed with unique molecule‐like properties due to discrete and complex electronic transitions.^[^ ^84^ ^]^ Metal NCs are often considered suitable for fluorescent dyes due to their good stability, the large Stokes shift, and adjustable excitation and emission spectra.^[^ ^85^, ^86^ ^]^ However, NCs have a low QY and unclear structure.^[^ ^87^ ^]^ To address these issues, enormous Au NCs have been reported in succession as NIR agents.^[^ ^88^, ^89^, ^90^ ^]^ In 2010, Wu described an imaging method by utilizing Au NCs stabilized by bovine serum albumin,^[^ ^91^ ^]^ which made it possible for Au NCs to be used as novel contrast agents. Through the analysis of mouse weight data, no obvious change was observed in the body weight of the experimental group injected with Au NCs, revealing that there was no toxicity problem with Au NCs. In 2011, Wang exhibited specific targeting and localization in HER2‐overexpressing breast cancer cells and tumor tissue due to conjugating Herceptin with Au NCs stabilized by bovine serum albumin (Au NCs‐Her).^[^ ^92^ ^]^ In 2014, our team synthesized the radiosensitizer GSH‐Au_25_ NCs (∼2.4 nm), which could significantly reduce tumor volume.^[^ ^93^ ^]^ In 2019, atomic‐precision gold clusters (Au_25_ (SG)_18_) were designed by our team (Figure 3D). Au_24_M_1_(SG)_18_ NCs (M = Cu) showed a QY of 0.67% in the case of standard IR‐26 dye with a QY of 0.05% as a reference sample. The fluorescence brightness of Au_25_ NCs can also be manipulated by ligand replacement techniques, such as glutathione, cysteine, and homocysteine. It is concluded that the mechanism of fluorescence enhancement is the change of electron transfer. Bright Au_25_ (SG)_18_ NCs could achieve deep tissue depth of penetration and be used for in vivo bioimaging detection. Furthermore, there was no neurotoxicity at a dose of 500 mg kg^−1^.^[^ ^55^, ^94^ ^]^ Other studies have also shown that Au_25_(SG)_18_ clusters can be exploited as an efficient fluorophore for bone imaging.^[^ ^95^ ^]^ In 2021, the CD‐Au NC labeled with anti‐CD326 antibody (Ab@Au NCs) could be efficiently tracked in vivo during blood circulation without interfering with biodistribution and tumor‐targeting capabilities, enabling sensitive tumor‐targeted imaging.^[^ ^96^ ^]^ A great nanocluster with a QY (∼8%) based on a rod‐shaped bi‐icosahedral [Au_25_ (PPh_3_)_10_(SC_2_H_4_Ph)_5_Cl_2_]^2+^ has also been developed, which has a peak emission wavelength of 1520 nm at the NIR‐II window.^[^ ^97^ ^]^

Copper and silver have also been proposed as promising alternatives.^[^ ^84^, ^98^ ^]^ Ag NCs have been developed for cancer cells imaging and subcutaneously xenografted tumors in vivo.^[^ ^99^ ^]^ A DNA‐stabilized silver nanocluster (DNA‐Ag NC) with an emission of approximately 960 nm in solution has been reported.^[^ ^100^ ^]^ Nevertheless, the emission wavelengths of Ag NCs are generally less than 1000 nm. Therefore, the imaging was mainly concentrated in the NIR‐I region. Although inorganic nanomaterials show great potential for NIR‐II imaging, there is still a big challenge due to long‐term toxicity and poor biocompatibility.

### 2.5 SMDs

**FIGURE 4:**
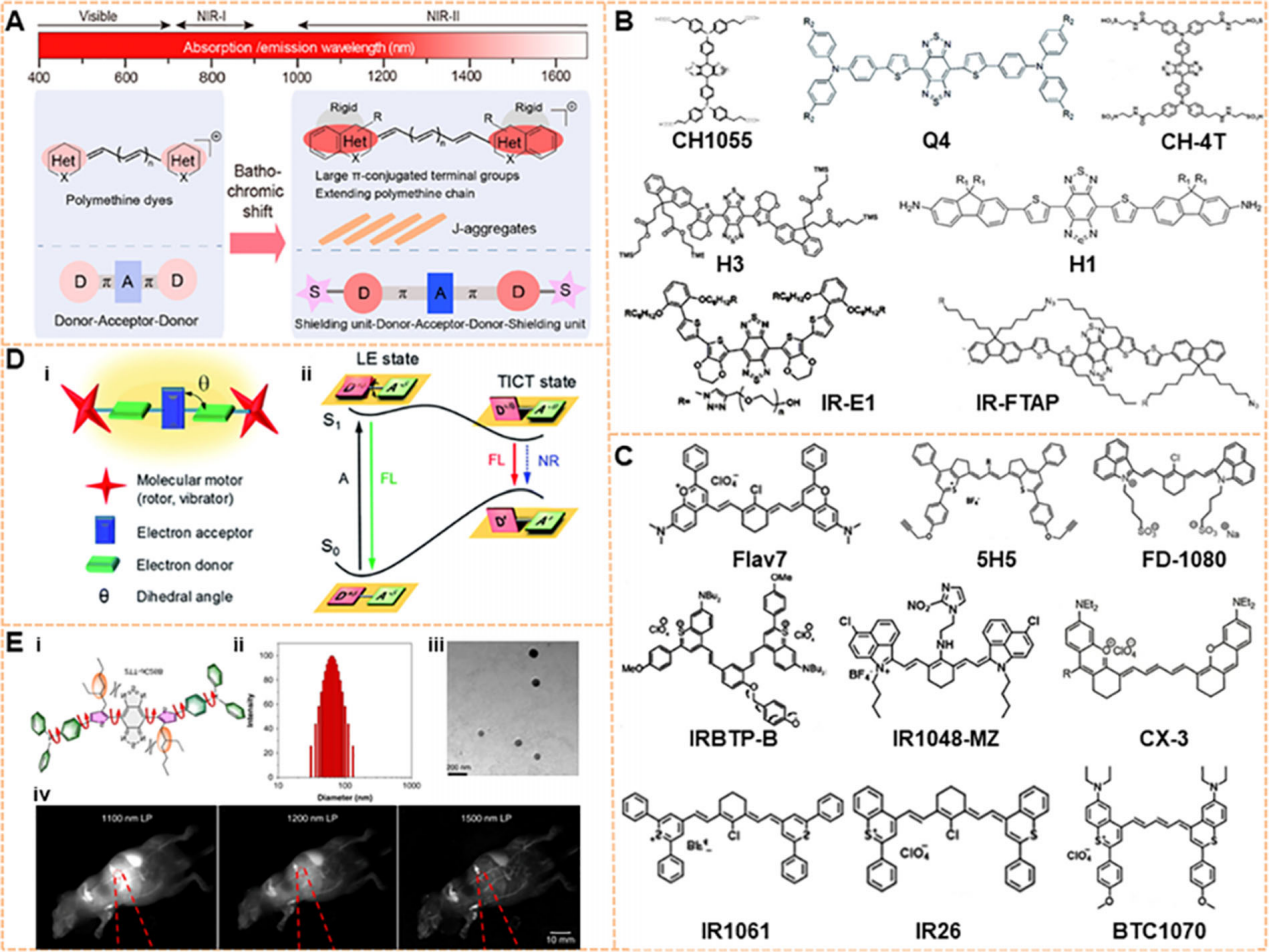
Organic fluorophores for NIR‐II imaging. (A) Design principles for organic NIR‐II fluorescent dyes. Reproduced with permission.^[^ ^51^ ^]^ Copyright 2020, American Chemical Society. (B) Chemical structure of D‐A‐D dyes with NIR‐II luminescence. Reproduced with permission.^[^ ^106^, ^107^, ^108^, ^111^, ^112^ ^]^ Copyright 2015, Springer Nature (CH1055); Copyright 2016 Royal Society of Chemistry (Q4); Copyright 2017, Royal Society of Chemistry (H1 and H3); Copyright 2017 Springer Nature (CH‐4T); Copyright 2018 American Chemical Society (IR‐E1 AND IR‐FTAP). (C) Chemical structure of polymethylene dyes with NIR‐II luminescence. Reproduced with permission.^[^ ^52^, ^122^, ^123^, ^124^, ^125^, ^128^ ^]^ Copyright 2013, American Chemical Society (IRBTP‐B and IR1048‐MZ); Copyright 2017, John Wiley & Sons (Flav7 and IR‐26); Copyright 2018, American Chemical Society (5H5); Copyright 2019, John Wiley & Sons (CX‐3); Copyright 2018, John Wiley & Sons (FD‐1080); Copyright 2013, John Wiley & Sons (IR1061 and BTC1070). (D) Molecular design strategies for AIEs with NIR‐II luminescence and Jablonski diagrams of TICT. Reproduced with permission.^[^ ^59^ ^]^ Copyright 2021, Royal Society of Chemistry. (E) Structure, size, and imaging of 2TT‐o C26B NP. Reproduced with permission.^[^ ^101^ ^]^ Copyright 2020, Springer Nature.

Many organic NIR‐II fluorophores with analogous function, good water‐soluble, and great biocompatibility have been studied simultaneously to solve these persistent problems in recent years, including SMDs,^[^ ^52^ ^]^ SP NPs,^[^ ^60^ ^]^ and AIEgens.^[^ ^101^ ^]^ These fluorophores have high fluorescence QY values, flexible functions, and adjustable luminescence spectra.^[^ ^102^, ^103^, ^104^, ^105^ ^]^ To date, SMDs are the most common luminescence group in fluorescent imaging. There are two main design principles for organic NIR‐II fluorophores at present: the first is the molecular structure containing donor‐acceptor‐donor (D‐A‐D). The electron donor and central receptors can generate strong electron movement, resulting in a low energy gap. The energy gap can be adjusted by the modified substantial donor and receptor groups so that the dye molecule can emit NIR‐II fluorescence. The bridging part of the π bond and the functional groups affect the QY by enhancing the intramolecular charge transfer and the rigidity of the molecule. Antaris and coworkers developed the NIR‐II organic small‐molecule base on the D‐A‐D structure for the first time, referred to as CH1055, which is the benefit for further development and reconstruction of the fluorescence imaging (Figure 4A).^[^ ^106^ ^]^ The carboxyl acid group of CH1055 was PEGylated by EDC/NHS chemistry, which further increased the solubility. Preliminary cytotoxicity tests have shown that the toxicity of CH1055‐PEG was not observed at higher doses, but the long‐term toxicity is unknown. PL excitation mapping of CH1055‐PEG demonstrated an excitation wavelength and an emission wavelength, showing a tail prolonging into the NIR‐II area.^[^ ^24^ ^]^ Figure 4B shows the structure of D‐A‐D dyes with luminescence at the NIR‐II window.^[^ ^106^, ^107^, ^108^, ^109^, ^110^, ^111^, ^112^ ^]^ With a distinguishing core structure from CH1055, the Q4 was developed on a D‐A‐D‐based basis by adding thiophene spacers with electron‐rich, showing excellent physical properties. Other small molecules can also be modified for functionalization, such as CH‐4T with sulfonic acid groups, H3 with four modifiable carboxyl groups, H1 with high photostability, IR‐E1 with thiophene‐based units, IR‐FTAP with the PEG_600_ version. Based on the design principles of D‐A‐D dyes, various NIR‐II fluorophores have been designed to reduce the structural gap. For instance, strong donor groups, such as fluorene, thiophene, and their products, have been introduced into the structure.^[^ ^16^, ^61^, ^107^, ^109^, ^113^ ^]^ The second design principle is based on the regulation of the structure of the polymethylene dye, which can achieve the purpose of red‐shifting the emission wavelength by increasing the length of the conjugate chain.^[^ ^52^ ^]^ To date, many polymethylene dyes with NIR‐II luminescence have been developed.^[^ ^114^, ^115^, ^116^, ^117^, ^118^ ^]^ including ICG. The main emission peak of ICG is at the NIR‐I window, and it can be tailed to the NIR‐II region.^[^ ^119^ ^]^ Further studies have indicated that the bright tail emission can yield high‐performance NIR‐II imaging.^[^ ^120^, ^121^ ^]^ Figure 4C shows the structure of currently available polymethine dye with NIR‐II luminescence.^[^ ^51^, ^122^, ^123^, ^124^, ^125^, ^126^, ^127^, ^128^, ^129^ ^]^ The optical properties of polymethine dyes with high absorption coefficient and mutable structural modification can be well improved. The absorption and emission peaks of some polymethine dyes, such as Flav7, 5H5, FD‐1080, IR1061, IR26, CX‐3, and BTC1070, can be controlled by extending the conjugated chain and adjusting the terminal group. The multifunctional molecular probe IR1048‐MZ conjugated with nitroimidazole achieves the centimeter‐level tissue depth penetration with high contrast. Therefore, these organic dyes are easily designed to be excellent biocompatibility and high quantum yield fluorescent dyes by introducing groups or other units.

At present, researchers have introduced the concept of AIE into the structural regulation of NIR‐II fluorescence molecules, which can resolve the issue of fluorescence quenching.^[^ ^56^ ^]^ However, most of the AIEgens are SMDs. As shown in Figure 4D, researchers designed AIEgens with a conjugated D‐A structure to lengthen the emission wavelength. The locally excited state can be transformed into the twisted intramolecular charge transfer (TICT) state because of the internal rotation of the D‐A unit in a polar environment. TICT caused a redshift of the emission wavelength while maintaining excellent photothermal performance. The interaction between molecules could be further prevented from triggering the AIE effect by introducing the molecular motor into the structure to obtain NIR‐II/AIEgens. The fluorescence intensity of AIEgens is significantly increased when they are in an aggregated state. In 2020, Tang and his group developed the organic fluorescent dye 2TT‐oC6B NP with NIR‐II emission.^[^ ^57^ ^]^ 2TT‐oC6B NPs possessed a high fluorescence QY at the molecular and morphological levels due to the TICT manipulation and the AIE effects of molecular distortion. The emission spectrum of 2TT‐oC6B NPs could be extended to 1600 nm. Moreover, the whole NIR‐II band (1000–1600 nm) showed a QY of 11.5% and the NIR‐IIb band (1500–1600 nm) exhibited that of 0.12% (Figure 4E),^[^ ^101^ ^]^ which inspired the development of NIR‐II dyes with longer wavelengths and higher brightness. The non‐toxicity of 2TT‐oC6B has been demonstrated by evaluating the cytotoxicity of human bladder cancer cells T24 and human urine epithelial cells. In 2022, a series of novel zwitterionic fluorophore with AIE properties at the NIR window have been rationally developed by Tang's group,^[^ ^130^ ^]^ showing the perfect performance in imaging and guiding the collaborative phototherapy against cancer. The routine examination and hepatic/renal function analysis of the mouse have demonstrated the negligible toxicity of AIE.

Although NIR‐II/AIEgens show good optical properties, some problems still need to be solved for further development. First, the overall brightness of AIEgens can be destroyed due to the skeleton distortions; Thus, it is still a challenge to maintain a high QY and increase the molecular absorption rate. Second, AIEgens displayed a short emission wavelength, which was not substantially larger than that of the NIR‐IIb region of a pure organic fluorophore. Third, the design of NIR‐II/AIEgens with biological functional is critical. Finally, the accumulation of NIR‐II/AIEgens can be found frequently in single nuclear phagocytic systems. Therefore, it is extremely critical to develop the renal clearance NIR‐II/AIEgens.

### 2.6 OSNs

OSNs have attracted more and more attention in the biomedical field and developed rapidly as an emerging class of fluorescent probes in the NIR‐II window.^[^ ^131^, ^132^, ^133^ ^]^ OSNs are designed from organic semiconducting polymers. Thus, OSNs with alternating donor and acceptor moieties consisting of highly π‐conjugated structures possess a sharper and stronger absorbance in the NIR‐II region.^[^ ^134^, ^135^ ^]^ Composed of organic and biologically inert components, OSNs show good biocompatibility with no heavy metal ions. More importantly, compared with some inorganic nanomaterials, OSNs display superior properties that show great promise to fluorescent nanomaterials, such as durable photostability, high extinction coefficient, and large Stokes shift. Moreover, the optical properties of OSNs can be manipulated by tuning chemical structure, functional groups, and size.^[^ ^136^ ^]^ The molecular versatility of OSNs plays a crucial role in regulating the photophysical properties. Therefore, OSNs have great potential for various biomedical applications in the NIR‐II window.^[^ ^137^ ^]^

In 2018, Bian's group designed and synthesized OSNs for photoacoustic (PA) imaging and stem cells tracking at the NIR‐II window.^[^ ^138^ ^]^ An OSN with a positively charged surface (OSPNs^+^) was developed by a “layer‐by‐layer modification” approach. The initial semiconducting polymers with hydrophobicity were encapsulated by using amphiphilic poly and then modified by Poly(_L_‐lysine) (PLL). The cellular uptake can be enhanced and efficient PA labeling of the stem cells can also be achieved by using the OSPNs^+^. In addition, the OSPNs^+^ showed excellent biocompatibility, suitable size, and optimization surface properties. The safety of the OSPNs^+^ was evaluated by performing the H&E staining, displaying excellent biocompatibility with no significant toxicity. In 2019, the first series of metabolizable OSNs for PA imaging were presented by Pu'group. The NIR‐II PA agents are easily degraded by myeloperoxidase and lipase degradation in abundant phagocytes, which contributes to the transformation from nonfluorescent nanoparticles (30 nm) into NIR fluorescent ultrasmall metabolites (≈1 nm), accelerating excretion without toxicity. The good biodegradability and high biosafety can be verified by SPN‐PT, SPN‐OT, and SPN‐DT. In 2020, Yin and colleagues reported novel amphiphilic OSNs with strong absorption in NIR‐II, enhancing the structural stability.^[^ ^33^ ^]^ The uniform morphology of OSNs could be formed by a self‐assembly process. The structural stability of OSNs promotes effective phototherapy for tumors at the NIR‐II window. A theranostic system based on OSNs (L1057) was reported by Yang and colleagues in 2020.^[^ ^50^ ^]^ The higher NIR‐II brightness facilitates the real‐time monitoring of the brain as well as the whole body and was successfully used to visualize cerebral ischemic stroke and tumors. In 2021, Yin et al. reported a self‐brightened OSNs to improve NIR‐II phototheranostics of the tumor by merging a weak electron‐donating unit into the semiconducting backbone to form the strong and alternating electron donor‐acceptor structure.^[^ ^139^ ^]^ In 2022, a nanomodulator with an immunoregulator was proposed by He and colleagues based on semiconductor polymer.^[^ ^140^ ^]^ The nanomodulator‐mediated combined photodynamic immunotherapy reboots the tumor immune microenvironment, producing highly effective tumor restraint and inhibition of lung metastasis.

## 3 NIR‐II IMAGING OF TUMOR HETEROGENEITY

The tumor is considered a highly heterogeneous and dynamic disease and is characterized by multiple molecular pathways during its evolution. During tumor progression, tumor heterogeneity arises from the tumor cells and the tumor microenvironment, which consists of immune cells, mesenchymal stem cells, endothelial cells, and fibroblasts.^[^ ^141^ ^]^ Researchers have investigated tumor heterogeneity by NIR‐II imaging in several studies.^[^ ^142^, ^143^, ^144^, ^145^, ^146^, ^147^, ^148^ ^]^ Several factors should be considered when developing probes for imaging tumor heterogeneity. The tumor‐specific NIR‐II fluorophores are designed to classify the heterogeneity and track the spatial location of tumors. For instance, CH1055 modified with tumor‐homing peptide was empowered to diagnose transplantable and spontaneous breast tumors.^[^ ^149^ ^]^ Thermal stable RNase A@ PbS/ZnS quantum dots were fabricated to visualize temporal changes of microvasculature remodeling in the NIR‐IIb region.^[^ ^150^ ^]^ A heptamethine‐cyanine‐based NIR‐II fluorophore SH1 can image the heterogeneity with intrinsic targetability.^[^ ^151^ ^]^ NIR‐II imaging shows the ability to delineate the inter‐ and intra‐tumor heterogeneity, which can be used to detail tumor histopathology.

### 3.1 NIR‐II imaging for inter‐tumor heterogeneity

**FIGURE 5:**
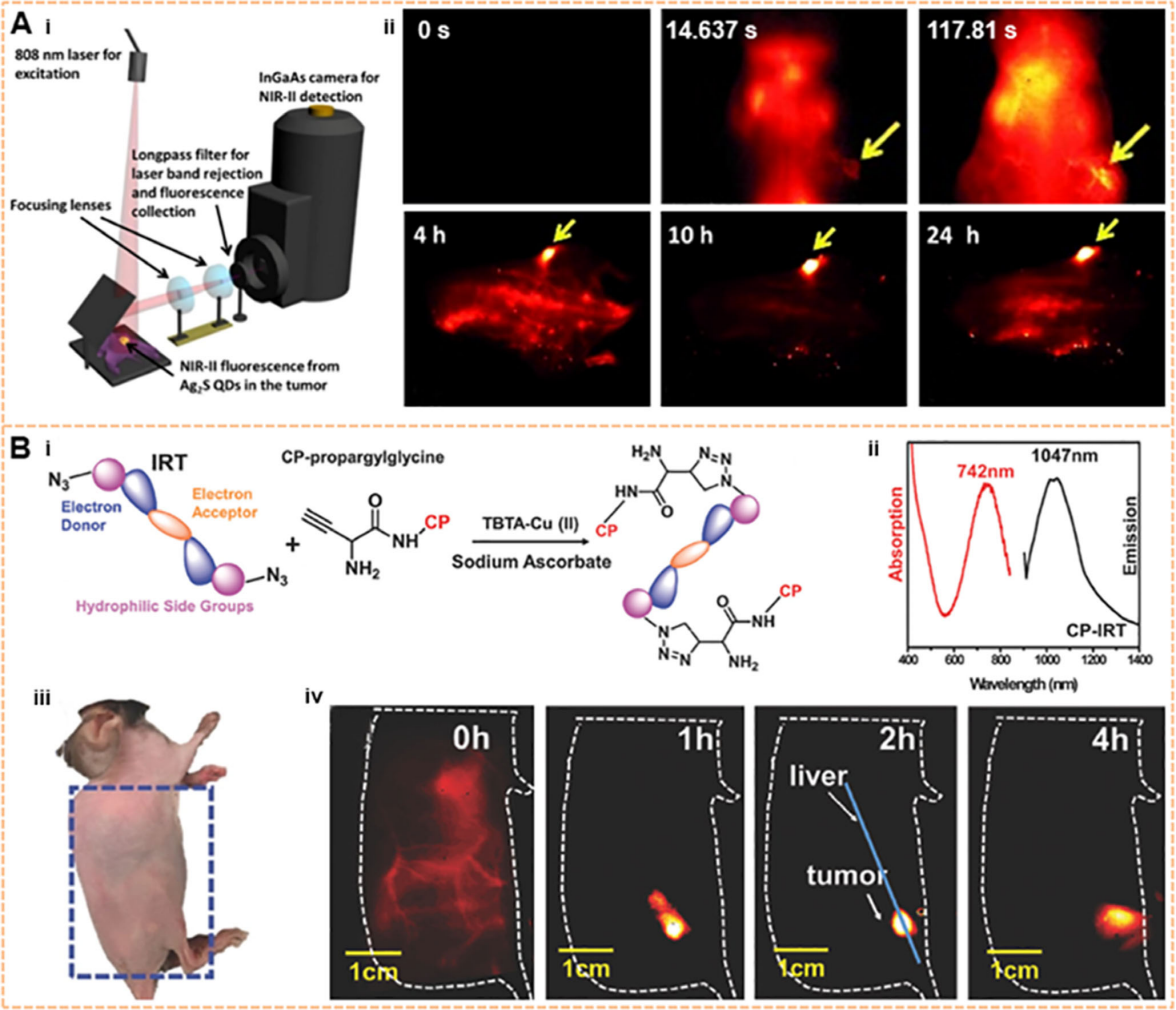
Image of near the skin surface tumor on the NIR‐II window. (A) Ag_2_S QD subcutaneous breast cancer imaging based on the EPR effect. Reproduced with permission.^[^ ^63^ ^]^ Copyright 2012, John Wiley & Sons. (B) Molecule structure, absorption spectra, and emission spectra of CD133 of the NIR‐II dye peptide probe CP‐IRT and its targeted imaging in mice subcutaneous colorectal cancer in vivo. Reproduced with permission.^[^ ^165^ ^]^ Copyright 2018, John Wiley & Sons.

Fluorescence imaging has the ability to identify the tumors’ locations near the skin surface in various cancers.^[^ ^152^, ^153^ ^]^ The NIR‐II fluorescence imaging of subcutaneous tumors can be achieved by the EPR effect. For example, in 2012, a two‐dimensional InGaAs array detector is used to image subcutaneous breast cancer by injecting 6PEG‐Ag_2_S, compared with visible light. As shown in Figure 5A, subcutaneous breast cancer could be accurately located within 4–24 h after injection.^[^ ^63^ ^]^ Chirality sorted (6, 5) carbon nanotubes at ultralow doses have also been used for breast cancer imaging.^[^ ^154^ ^]^ Breast cancer and the vascular structures around tumors have been explored in a live mouse by using semiconducting SWNTs at the NIR‐IIb window.^[^ ^155^ ^]^ A metabolizable SPN‐PT has been developed for PA imaging of subcutaneous tumors.^[^ ^49^ ^]^ Subsequently, with the development of tumor markers and the progression of conjugation technology, molecular imaging targeting specific tumor cells has become feasible. Breast cancer can be differentiated from surrounding normal tissues by using the folate receptor‐specific NIR‐II probe.^[^ ^156^ ^]^ Furthermore, the whole human breast can be targeted for NIR‐II photoacoustic imaging by loading the coenzyme Q10 into surfactant‐stripped CyFaP with a long absorption wavelength.^[^ ^157^ ^]^ AIEgens HLZ‐BTED dots have been employed in visualizing breast tumors and observing tumor‐feeding blood vessels, hind limb vasculature, and hind limb ischemia.^[^ ^158^ ^]^ Additionally, CuInSe_2_@ZnS QDs with a NIR‐II QY of 21.8% have been used to perform autofluorescence‐free bioassays of circulating human breast tumor cells in whole blood samples.^[^ ^159^ ^]^ Human squamous cell carcinoma (SCC) tumor models were precisely imaged by using CH1055 conjugated with epidermal growth factor receptor (EGFR).^[^ ^106^ ^]^ Then, Erbitux (Erb) and EGFR affibody were covalently conjugated onto IR‐FGP and then used to target‐image SCC to clearly distinguish between SCC and normal tissue via non‐irritant copper‐free click chemistry.^[^ ^160^ ^]^ Furthermore, an innovative molecular imaging probe can be obtained by conjugating the NIR‐II fluorophore SWCNT and streptavidin. Multiple color imaging of SCCs confirmed that the EGFR can be highlighted through IR‐FGP, vessels can be displayed through SWCNT, and the nucleus can be presented through a commercial fluorophore. This composite probe targeted the SCC and imaged the blood vessels surrounding the tumor, allowing further exploration of SCC development.^[^ ^61^ ^]^ The dual‐modal NIR‐II/MRI NaGdF_4_‐PEG‐cMBP nanoprobe detected early head and neck squamous cell carcinomas by targeting cMet receptors.^[^ ^161^ ^]^ In addition to SCC and breast cancer, NIR‐II targeted imaging is also possible for malignant melanoma. For example, diketopyrrolopyrrole‐based OSNs (PDFT1032) have been considered to be NIR‐II fluorophores for imaging tumors in a subcutaneous melanoma model.^[^ ^162^ ^]^ SWCNTs have been developed for triggered selective imaging of human melanoma by using anti‐GD2 antibody‐attached gold nanoparticle conjugation.^[^ ^163^ ^]^ In addition to forming tumor‐targeted probes with antibodies as binding objects described above, peptides have also been used in chemical reactions with organic solvents which are incompatible with antibodies to synthesize tumor probes. As a standard tumor marker, CD133 is overexpressed in many tumors, including colon cancer, liver cancer, and ovarian cancer.^[^ ^164^ ^]^ CD133 has been targeted by a peptide CP conjugated to an organic D‐A‐D dye IRT. The fluorescence emission peak of this CP‐IRT is located at ∼1050 nm with a QY of ∼1.5%. The CP‐IRT molecular probe has been used to image subcutaneous human colon cancer with T/NT ∼8.3 and could be rapidly cleared by the kidneys (Figure 5B).^[^ ^165^ ^]^

**FIGURE 6:**
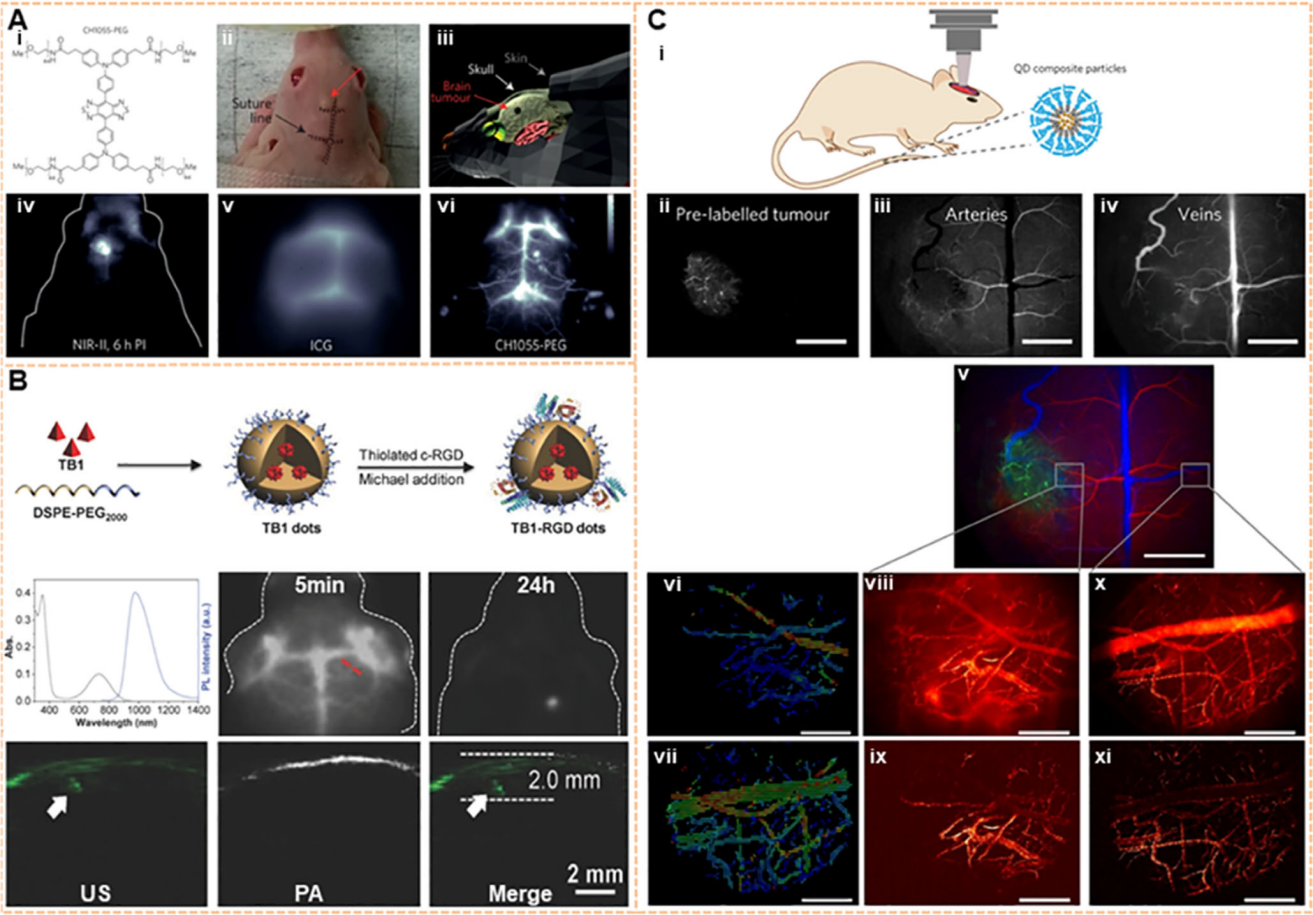
Image of the brain tumor on the NIR‐II window. (A) Through‐skull noninvasive NIR‐II imaging at the depth of 4 mm with CH1055‐PEG. Reproduced with permission.^[^ ^106^ ^]^ Copyright 2015, Springer Nature. (B) Preparation, absorption spectra, and emission spectra of TB1‐RGD dots; Fluorescence signals of brain tumor location inside brain tissues. Reproduced with permission.^[^ ^56^ ^]^ Copyright 2018, John Wiley & Sons. (C) The glioblastoma multiforme was targeted by InAs‐based QDs composite particles under the NIR‐II window. The vascular networks around the tumor and healthy brain tissue were mapped, and blood flow was measured quantitatively. Reproduced with permission.^[^ ^44^ ^]^ Copyright 2017, Springer Nature.

To date, brain tumors are still difficult to be diagnosed due to the complicacy of the central nervous system. Moreover, brain tumors are among the most lethal tumors due to the blood‐brain barrier (BBB) and the lack of efficient brain tumor treatment options. Non‐invasive NIR‐II fluorescence imaging achieved the 4 mm depth penetration in glioblastoma through the intact scalp and skull via intravenous injection of CH1055‐PEG with a T/NT ratio of 5.5. Meanwhile, the cerebral blood vessels in the NIR‐IIa region are clearly observed (Figure 6A).^[^ ^106^ ^]^ A NIR‐II molecular fluorophore based on the S‐D‐A‐D‐S structure was synthesized from IR‐BEMC6P, and then conjugated to an Arg‐Gly‐Asp (RGD) peptide that targeted α_V_β_3_ integrin receptors. IR‐BEMC6P@RGD probes have also been employed in performing noninvasive NIR‐II imaging of brain tumors with high‐performance and rapid renal excretion.^[^ ^166^ ^]^ TB1‐RGD dots, based on AIE dots, delineated the depth of the tumor in the brain, accurately assessing its location within the brain tissue (Figure 6B).^[^ ^56^ ^]^ PSY, which incorporates organoplatinum (II) metal cycle P1 and SY1030 into polymer Pluronic F127, can be internalized into glioma U87MG cells.^[^ ^167^ ^]^ In addition, the P1 RGD NPs assisted the clear localization of glioma in the scalp and skull with real‐time PA imaging with signal to background ratio (SBR) up to 90.^[^ ^168^ ^]^ NaNdF_4_ NPs were delivered into the brain by focused US to temporarily open the BBB. Thus, orthotopic glioblastoma was detected by fluorescence imaging at 1340 nm.^[^ ^169^ ^]^

Malignant brain tumors are characterized by histopathological features, including microvascular proliferation and pleomorphic vessels.^[^ ^170^ ^]^ Indium arsenide (InAs) QDs with QY up to 82% have been used to image the brain vasculature in mouse models.^[^ ^171^ ^]^ Subsequently, the glioblastoma multiforme was targeted by InAs‐based QD composite particles at the NIR‐II window by using one‐photon excitation SWIR intravital microscopy. Simultaneously, QD nanocomposite particles were used to visualize tumor growth and reveal the networked structure of blood vessels during tumor growth, showing irregular blood flow of the glioblastoma margin. In contrast, the healthy brain tissue showed a standard vascular network and regular blood flow. Thus, blood flow in the tumor margin and the healthy brain can be quantitatively analyzed by particle image velocimetry. Finally, abundant brain blood flow maps can be mapped within minutes (Figure 6C).^[^ ^44^ ^]^ Based on the results of these studies, accurate targeted imaging of glioblastoma multiforme has been carried out. Furthermore, the detailed biological information and pathological process of glioblastoma can be explored visually, which provides the possibility for future study of the pathogenesis of glioblastoma, surgical navigation, and therapy. Another brain tumor, medulloblastoma, is the most malignant intracranial glioma, which mainly affects children under the age of 14. Through‐scalp imaging has been achieved using *m*‐PBTQ4F polymer dots. The imaging results revealed that the medulloblastoma vasculature was unevenly distributed and chaotic. Furthermore, the vasculature exhibited serpentine courses and irregular branches. These findings offer insightful information for the correct diagnosis of medulloblastoma.^[^ ^172^ ^]^ However, prompt treatment of early‐stage microscopic tumors with a diameter less than 2 mm is beneficial for prolonging patient survival before angiogenesis. Most studies have focused on brain tumor imaging of advanced tumors with bulky volume and compromised blood‐brain barrier. Due to the lack of permeability of BBB, the precise diagnosis of tumors under the microscope remains challenging. Temporary and secure BBB opening with temporal and spatial specificity can be provided by combining the focused US with microbubbles. The precise position of microscopic brain tumors has been evaluated by using PBT NPs designed with dual NIR‐II signal and a high SBR of 7.2.^[^ ^173^ ^]^ In addition to the above studies on brain tumors in the NIR‐II range, several other studies have been conducted.^[^ ^174^ ^]^ For example, brain tumors have been targeted‐imaged by the small‐molecule NIR‐II dye SHX,^[^ ^108^ ^]^ perylene‐3,4,9,10‐tetracarboxylic diimide (PDI) NPs,^[^ ^175^ ^]^ and conjugated polymer nanoparticles.^[^ ^176^ ^]^ Recently, a DNA block copolymer with a peak at 1040 nm, PS‐b‐DNA, was designed by a solid‐phase click reaction, which could cross the BBB by using the receptor‐mediated transcytosis pathway.^[^ ^177^ ^]^ This represents an excellent tool for future noninvasive learning of brain tumors.^[^ ^178^ ^]^

**FIGURE 7:**
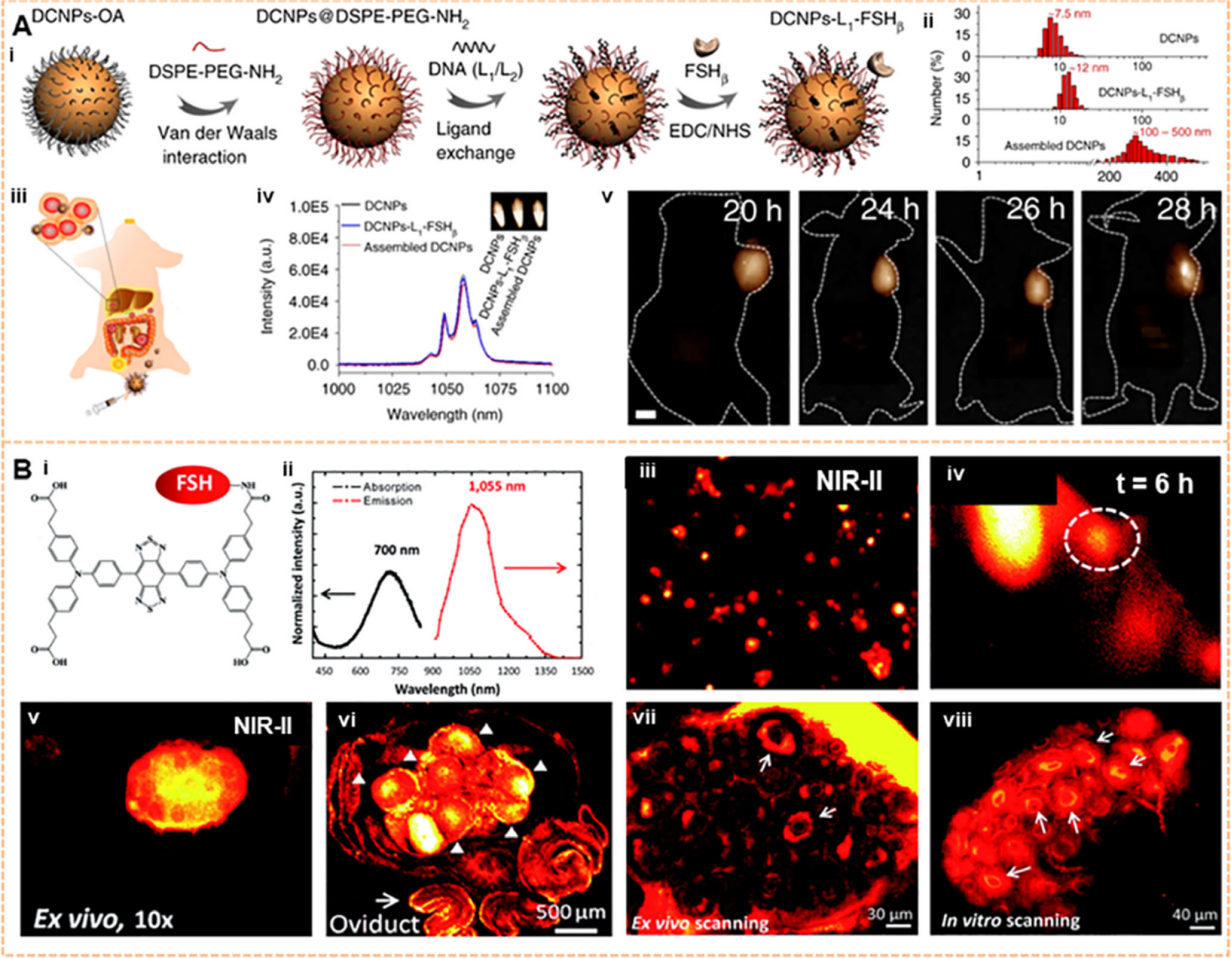
Image of the ovarian tumor on the NIR‐II window. (A) The DCNPs‐L1‐FSHβ nanoprobes accurately targeted ovarian serous carcinoma. Reproduced with permission.^[^ ^180^ ^]^ Copyright 2018, Springer Nature. (B) Ovarian follicles granulosa cells of antral/smaller follicles were found in vivo by injecting an FSH‐CH probe. Granulosa cells of secondary follicles (arrows) were also clearly visualized ex vivo and in vitro using a confocal fluorescence microscope. Reproduced with permission.^[^ ^187^ ^]^ Copyright 2017, Royal Society of Chemistry.

Ovarian cancer has the highest mortality rate among gynecologic malignancies.^[^ ^179^ ^]^ In particular, serous ovarian carcinomas are typically diagnosed at an advanced stage within the peritoneal cavity. DCNP‐L1‐FSHβ nanoprobes with a T/N ratio higher than 9.0 can be used to diagnose early serous ovarian cancer (Figure 7A).^[^ ^180^ ^]^ An organic dye (IR1061), SWNT, PbS, and DCNP (NaY_0.78_Yb_0.2_Er_0.02_F_4_) have been assembled into a NIR‐II probe by a layer‐by‐layer system.^[^ ^78^ ^]^ Many investigations have also been performed for the early diagnosis and screening of ovarian cancer at the NIR‐II window.^[^ ^181^, ^182^, ^183^ ^]^ Ovarian tumors could be detected early by utilizing lanthanide DCNPs (Er^3+^‐ and Ho^3+^–doped).^[^ ^78^, ^182^ ^]^ Probe BOD‐M‐βGal with NIR‐II fluorescence revealed the fast and precise imaging of ovarian tumors.^[^ ^181^ ^]^ Targeted M13 virus‐stabilized SWNTs (SBP‐M13‐SWNTs) have been used to image ovarian tumors, thereby improving the noninvasive detection of tumors and providing guidance for the surgical removal of submillimeter tumors.^[^ ^184^ ^]^ In addition to the early diagnosis of primary ovarian cancer, NIR‐II imaging is also of great value for detecting distant metastasis of ovarian cancer. After intravenous injection of NIR‐II Pdots, whole‐body organs, vessels, and peritoneal and lymphatic metastases of ovarian cancer can be visualized by NIR‐II fluorescence imaging.^[^ ^185^ ^]^ By using a self‐assembly NIR‐II confocal system, the 3D mapping of whole ovaries has been reported without the use of tissue‐clearing techniques. This approach reached an excellent 900 μm scanning depth after labeling with an IR‐FEPC.^[^ ^186^ ^]^ Another study showed that ovarian follicle granulosa cells of antral and smaller follicles could be detected by injecting an FSH‐CH probe. Furthermore, this research group observed the ovaries containing only preantral or smaller follicles. Secondary follicle structures were clearly shown by using high‐magnification confocal fluorescence imaging (Figure 7B).^[^ ^187^ ^]^ These high‐resolution imaging studies of ovarian structure demonstrated the value of the aforementioned tools for further studying the heterogeneity and development of ovarian cancer.

### 3.2 NIR‐II imaging of tumor microstructure

**FIGURE 8:**
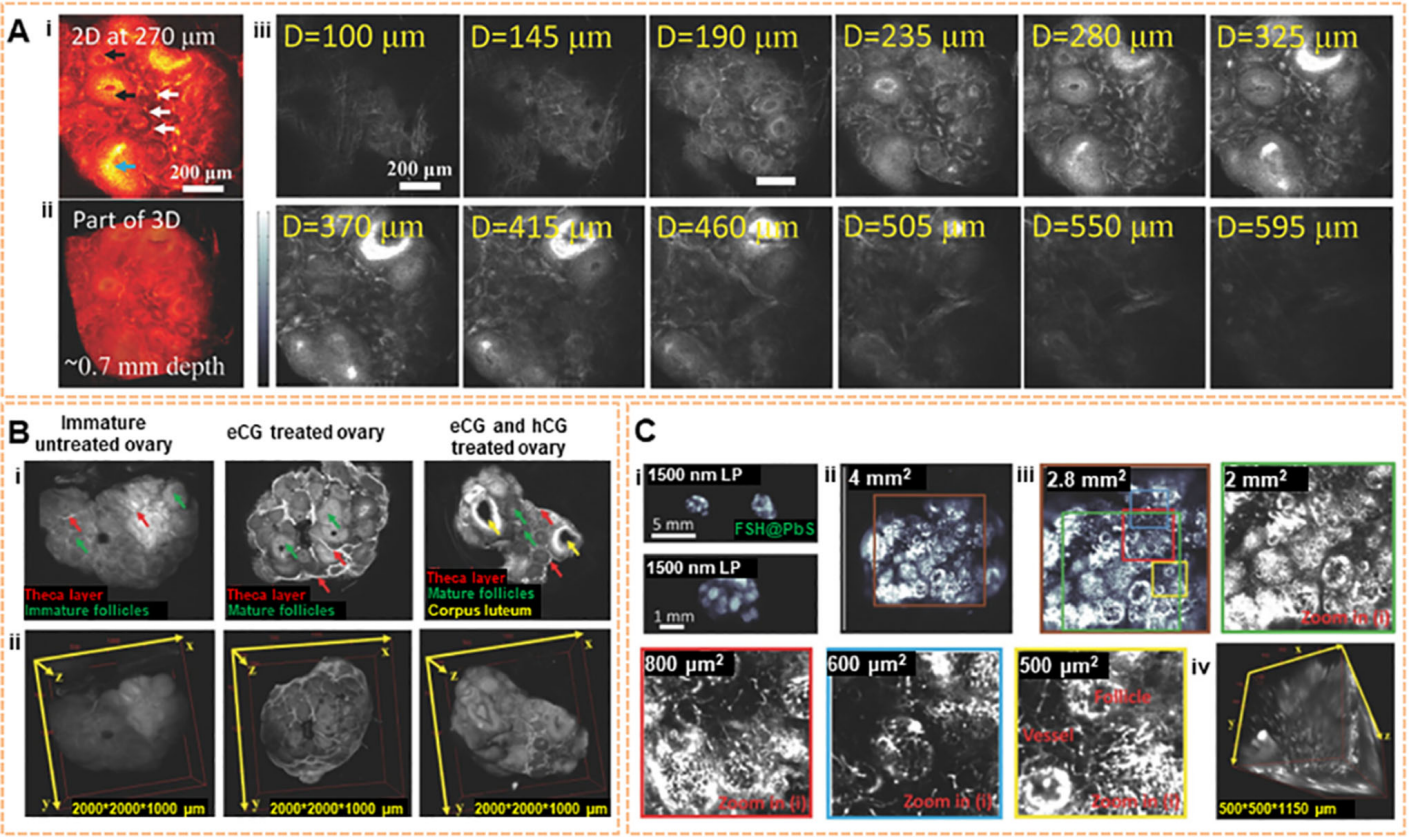
2D and 3D imaging of mouse ovary microstructure using IR‐FEPC. Reproduced with permission.^[^ ^186^ ^]^ Copyright 2018, John Wiley & Sons. (A) Confocal image of a portion of the ovary. The signals of theca (white arrows), granulosa cells (black arrows) inside individual follicles, and corpora lutea (blue arrows) (excitation: 785 nm, emission: 1100 nm). (B) Three NIR‐II imaging results: the theca layer in ovarian follicles, preovulatory follicle mature granulosa cells, and corpus luteum by using hCG@IR‐FEPC. (C) Follicles and vessels of the ovary were magnified imaging.

Researchers and clinicians visualize deeper areas of the body with high‐clarity NIR‐II macroscopic/microscopic imaging.^[^ ^188^ ^]^ Ag_2_S QDs achieved a higher resolution image of deep tumor structures and directly highlighted the tumor vascular structures.^[^ ^189^ ^]^ In particular, the use of confocal imaging and 3D reconstruction with NIR‐II imaging makes it easier to visualize and understand the microstructure inside tumors. As shown in Figure 8, using luteinizing hormone (LH) as a model, the mouse ovary was targeted for imaging. IR‐FEPC has been conjugated to human chorionic gonadotropin (hCG) for specific imaging of the 3D structure of the ovary by using a one‐photon confocal microscope. On this basis, the process of gonad development has been studied to elucidate the vital role of hCG in ovarian development and to reveal the hCG receptor state at different periods of ovary development. More crucially, 2D and 3D confocal imaging of the ovary stained by FSH@PbS has also revealed specific follicular and vascular structures at a depth of approximately ∼1125 μm with micrometer resolution.^[^ ^186^ ^]^ These findings provide the basis for examining in vivo the LH receptors in the uterine at different physiological periods in the future. These methods can be used to dynamically monitor the disease progression of Leydig cell tumors by detecting LH secretion in normal Leydig cells.

**FIGURE 9:**
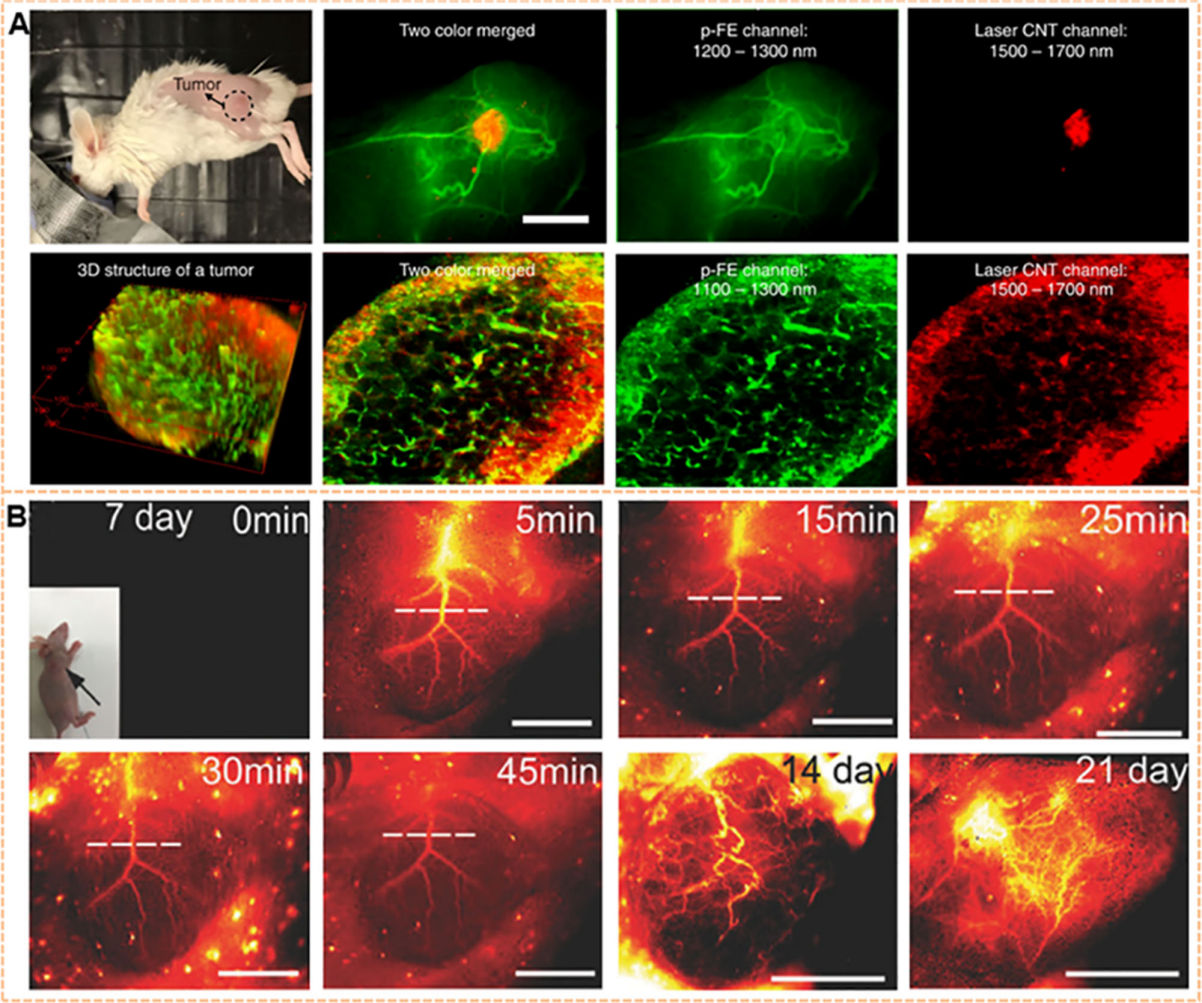
Imaging of tumor microstructure. (A) Confocal imaging of tumors by using two channels at the NIR‐II window. 740 μm × 740 μm × 220 μm area, step size: 2, 2, 5.4 μm with *x*, *y, z* directions. scanning speed: 15 min/frame, laser power: ∼30 mW, PMT voltage: 500 and 600 V for p‐FE channel and laser CNT channel, respectively. Scale bars represent 6 mm. Reproduced with permission.^[^ ^191^ ^]^ Copyright 2018, Springer Nature. (B) Dynamic assessment of angiogenesis of tumors in vivo by using CQS1000. The disorganized and tortuous pattern of the blood vessel in osteosarcoma was shown. Reproduced with permission.^[^ ^16^ ^]^ Copyright 2017, John Wiley & Sons.

Tumor angiogenesis is essential for tumor progression. NIR‐II fluorophores have been widely used for ultrafast imaging of blood vessels and monitoring of tumor vessels in vivo.^[^ ^190^, ^191^, ^192^, ^193^, ^194^, ^195^, ^196^, ^197^ ^]^ Chirality‐enriched (12,1) and (11,3) SWNTs as NIR‐II contrast agents have been described for xenograft tumor angiography. Moreover, the minimum resolvable vessel width is < 10 μm.^[^ ^198^ ^]^ An RGD‐based NIR‐II fluorescent probe has been used for imaging the tumor vasculature.^[^ ^199^ ^]^ Tumor vascular structure can be directly observed and non‐invasively assessed by utilizing NIR‐II Ag_2_S QDs.^[^ ^189^ ^]^ The Dai group successfully evaluated a mouse tumor microstructure in vivo indicating the exciting potential for monitoring and understanding tumor behaviors in vivo. Fluorescence signals of tumors from p‐FE emitting and laser CNTs emitting can be extracted at the NIR‐II window, realizing two‐color imaging, highlighting rich blood vessels around and inside the tumor, and visualizing the highly aggressive biological behavior of the tumor. Additionally, 3D layer‐by‐layer confocal imaging has been utilized to describe the detailed structure of tumor tissue in vitro, and it was found that there were more blood vessels around the tumor than inside the tumor (Figure 9A).^[^ ^191^ ^]^ As shown in Figure 9B, the condition of angiogenesis and the evolution of tumors have been visualized by using CQS1000. Furthermore, angiogenesis development and evolution could be explored as the tumor grew.^[^ ^16^ ^]^

In conclusion, NIR‐II fluorescence microscopy is considered to play an extremely important role in deep‐tissue molecular imaging, which provides a new solution for capturing detailed tissue structures.^[^ ^200^ ^]^

### 3.3 NIR‐II imaging for immunotherapy

Immunotherapy is defined as an emerging method to act on tumors by stimulating the patient's immune system to kill tumor cells. The blockade of the immune checkpoint targets regulatory pathways in T cells to improve antitumor immunoreaction, showing significant clinical benefits in different kinds of tumors. Tumor immune escape can be reversed by hindering the programmed death 1 (PD‐1) pathway with program cell death ligand 1 (PD‐L1) monoclonal antibodies. Meanwhile, powerful antitumor immunity can also be generated, resulting in durable tumor regression.^[^ ^201^, ^202^ ^]^ Therefore, predicting patient response to treatment, monitoring the process of complex immune cells in the microenvironment of living tumors, and evaluating heterogeneous changes in PD‐L1 within tumors are vital for improving the function of immune checkpoint blockade. Currently, the expression status of PD‐L1 in tumor cells is analyzed in vitro by immunohistochemistry, which relies on biopsy. PET can be performed to address the PD‐L1 distribution in vivo.^[^ ^203^, ^204^ ^]^ Due to the impressive advantages of enhanced penetration depth and improved SBR, NIR‐II imaging provides a potent tool for understanding the immune cell recognition of tumor cells and the mechanism of immune cell initiation and invasion. More crucially, NIR‐II imaging delineates the heterogeneity of immune cells in tumor tissue in vivo.

**FIGURE 10:**
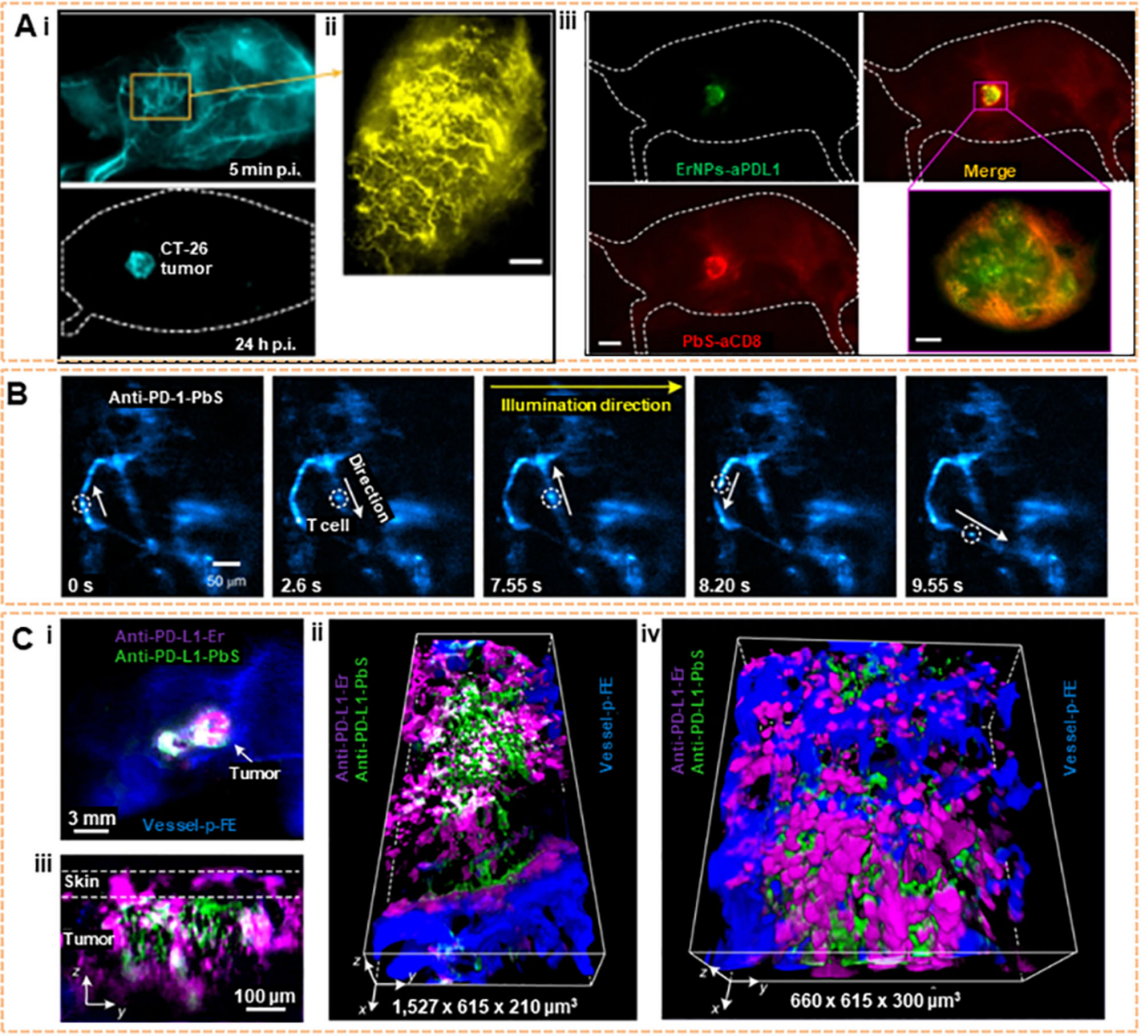
NIR‐II imaging for immunotherapy. (A) In the left margin, the wide‐field image of a CT‐26 tumor mouse injected with Er NPs‐1/10^th^ that PD‐L1 containing 20 μg anti‐PD‐L1 mAb (1 mg kg^−1^); High‐magnification molecular imaging of the CT‐26 tumor at 5 min post‐injection of Er NPs‐1/10^th^ a PD‐L1 (scale bar, 500  μm). In the right margin, the different heterogeneous signal distributions of PbS‐aCD8 and ErNPs‐aPDL1. Dual molecular imaging (upper right) of a CT‐26 tumor mouse injected with mixed ErNPs‐aPDL1 (green color, upper left) and PbS‐aCD8 (red color, lower left). Reproduced with permission.^[^ ^207^ ^]^ Copyright 2019, Springer Nature. (B) Time‐course recording of PD‐1^+^cells (white circles) in a CT26 tumor labeled by anti‐PD‐1 CSQDs at 20 frames per second by oblique LSM (excitation: 1319 nm; emission: 1500–1700 nm). Reproduced with permission.^[^ ^208^ ^]^ Copyright 2019, Springer Nature. (C) Wide‐field imaging of cells labeled with anti‐PD‐L1 ErNP (magenta), anti‐PD‐1 PEGylated PbS/CdS CSQD (green), and p‐FE filling vessels (blue) in a CT26 tumor (upper left). Reproduced with permission.^[^ ^208^ ^]^ Copyright 2019, Springer Nature.

Anti‐PD‐L1‐BGP6 can accurately discriminate the expression level of PD‐L1 in different cell lines in vitro and in vivo.^[^ ^205^ ^]^ BDP‐in‐anti‐PD‐L1 NPS actively targets colon cancer due to antibody binding to the receptor and simultaneously facilitates NIR‐II imaging.^[^ ^206^ ^]^ Er NPs with bright down‐conversion luminescence at 1600 nm have been used to dynamically image immunotherapy. Moreover, the process of Er NPs‐aPDL1 infiltration into tumor tissue from the blood vessels and its binding to tumor cells has been visualized. Additionally, multiplexed fluorescence imaging with ∼1600 nm emission has been performed by utilizing Er NPs and PbS with millisecond and microsecond lifetimes, respectively. Dual molecular imaging showed that CD8^+^ cytotoxic T lymphocytes (CTLs) in the tumor microenvironment respond to immunotherapy. CTLs were found to be activated in the spleen and migrated into the tumor to eliminate tumor cells. Critically, the heterogeneity of CD8^+^ CTLs within the tumor could be visualized by using these two probes to show that CD8^+^ CTLs were more distributed in the outer region of colon tumors than in the inner region (Figure 10A).^[^ ^207^ ^]^ Thus, NIR‐II fluorescence molecular imaging offers visualization, real‐time monitoring, and assessment of target sites of the immune checkpoint PD‐L1 in tumors. As shown in Figure 10B, dynamic imaging revealed a single PD‐1^+^ T cell irregularly in the colon cancer vasculature and a subsequent reversal of blood flow direction by using Er NPs and CSQD probes. PD‐1^+^ T cells extravasate from the blood vessels, resulting in the surrounding PD‐L1^+^ cancer cells in the tumor, which is a crucial process for starting immunotherapy. This process was visualized by using triple oblique NIR‐II light‐sheet microscopy to delineate the 3D spatial distribution of PD‐L1 (labeled by anti‐PD‐L1 Er NPs), PD‐1 (labeled by anti‐PD‐1 CSQD), and the blood vascular system (labeled by p‐FE) of the tumor (Figure 10C).^[^ ^208^ ^]^ The whole process of anti‐PD‐L1 tumor immunotherapy can thus be visualized in vivo through the NIR‐II window.

Moreover, many studies have exploited the advantages of NIR‐II to trigger tumor‐enhancing immunotherapy and found that it significantly affects the elimination of primary and metastatic tumors.^[^ ^209^, ^210^, ^211^, ^212^, ^213^, ^214^ ^]^ For example, an activatable polymer nanoagonist (APNA) effectively ablated a tumor located at a depth of 8 mm. Furthermore, the APNA enhanced cytotoxic T lymphocyte and helper T cell infiltration into a distal tumor, the lung, and the liver, thereby inhibiting tumor metastasis.^[^ ^215^ ^]^ NIR‐II PA/MR image‐guided PTT reversed immunosuppression.^[^ ^216^ ^]^ Under NIR‐II light irradiation, the membrane‐coated semiconducting polymer nanoengager eradicated the tumor and induced immunogenic cell apoptosis, inducing antitumor T cell immunity and acquiring immune memory.^[^ ^217^ ^]^ Catalytic immunotherapy based on artificial enzymes has shown promising efficacy in tumor immunotherapy.^[^ ^218^ ^]^ Engineering oxaliplatin prodrug nanoparticles has provided a promising approach for colorectal cancer treatment.^[^ ^219^ ^]^ CSP@interleukin‐12 was explored for tumor localized NIR‐II PTT and in situ immunotherapy through the local generation of the interleukin‐12 cytokine.^[^ ^211^ ^]^ To date, NIR‐II fluorescence imaging has successfully provided reliable monitoring of the location, dynamics, and function of the immune response. This imaging strategy is critical not only for evaluating efficacy but also for optimizing the safety and design of immunotherapy.

## 4 NIR‐II IMAGING OF TUMOR PROGRESSION

The tumor, node, and metastases (TNM) classification system is the most robust prognostic parameter for stratifying patients. The depth of tumor invasion through the tumor wall, the number of involved lymph nodes, and the presence of metastases can be unambiguously described by the TNM system. Therefore, tumors can be divided into 4 different stages with different prognostic and therapeutic consequences. T, N, and M are important prognostic indicators for cancer patients and essential factors in formulating a treatment plan. T, N, and M are also markers of tumor progression. Tumor progression has been monitored by recording tumor images and measuring the volume change with HA‐4‐ATP‐AuNFs at a wavelength of 1200 nm.^[^ ^220^ ^]^ Early small tumors triggered by 808 nm laser irradiation have been detected by PAA‐NRs,^[^ ^221^ ^]^ Nd‐DTPA,^[^ ^222^ ^]^ and RNase‐A@Au NCs.^[^ ^223^ ^]^ AIEgens (TQ‐BPN) can identify the tumor at different progression stages by utilizing NIR‐II fluorescence microscopy. After 24 h of injection with TQ‐BPN dots, the 4‐week subcutaneous xenograft tumor was not perfectly handled, while the 2‐week tumor was signally visualized. Furthermore, abundant fluorescence aggregated outside the vessels of the 2‐week tumor, in apparent contrast to that in the 4‐week tumor.^[^ ^224^ ^]^ Nd^3+^–doped core–shell NCs have been used to exhibit the evolution process of the tumor vascular system in the subcutaneous breast tumor during progression, tumorigenesis, growth, and necrosis.^[^ ^225^ ^]^ NaLnF_4_:Yb/Er@Cu_2‐x_S hybrid nanoprobes with a core‐satellite structure at 1525 nm emission are capable of detecting early tumors (5 mm in diameter).^[^ ^226^ ^]^ Therefore, NIR‐II fluorescence imaging can visualize the progression of tumors.^[^ ^227^ ^]^ Multimodal imaging, including CT, US, MRI, and NIR‐II, improved the accuracy of tumor staging by integrating different imaging modes into a hybrid system.^[^ ^132^, ^228^, ^229^, ^230^, ^231^, ^232^, ^233^ ^]^ NIR‐II PA tomography imaging showed that BTII‐DUPA can target actively the prostate‐specific membrane antigen‐positive prostate tumors in vivo.^[^ ^234^ ^]^ CH‐4T/SLB‐MSN‐Mdot/Cu^2+^ nanoprobes for PET/NIR‐II imaging could be applied for tumor detection and delineation.^[^ ^235^ ^]^ FS‐GdNDs with enhanced multifunctional characteristics displayed satisfactory dual‐modal MR/NIR‐II imaging performance at a relatively low dose.^[^ ^236^ ^]^ BDT‐TQE SP‐cored nanoparticles with TICT features exhibit a high NIR‐II photothermal conversion efficiency and realize long‐term monitoring of in situ hepatic tumor growth by PA/US dual‐modality imaging in vivo.^[^ ^237^ ^]^

### 4.1 NIR‐II imaging of lymph node metastases

**FIGURE 11:**
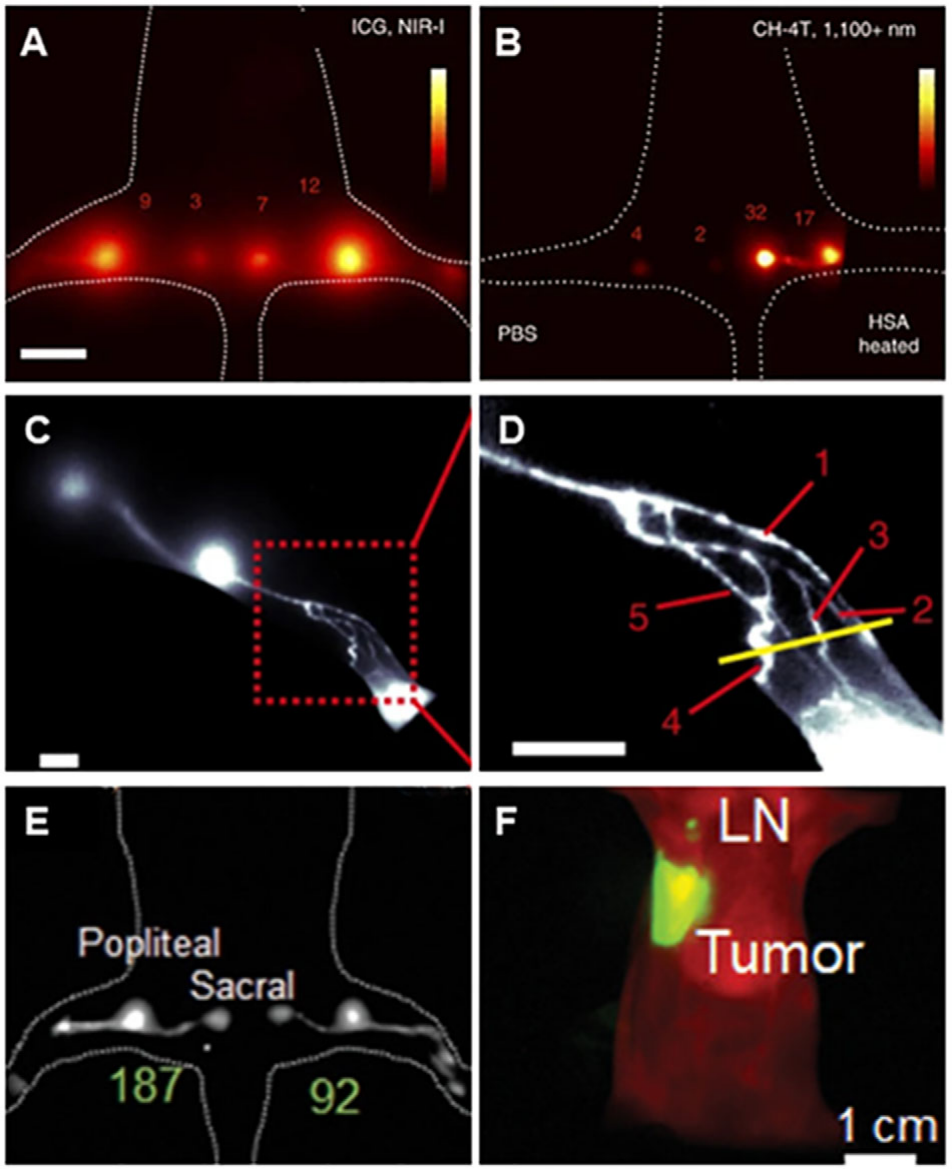
NIR‐II imaging of the lymph nodes. (A) NIR‐I fluorescent imaging of popliteal and sacral lymph nodes by using 50 μM ICG (exposure time: 50 ms; color bar: 4000 to 40,000; ∼30 min post‐injection). Reproduced with permission.^[^ ^111^ ^]^ Copyright 2017, Springer Nature. (B) NIR‐II fluorescent image of popliteal and sacral lymph nodes by using 50 μM CH‐4T/PBS (in the left foot) and CH‐4T/HSA‐HT (in the right foot) at equivalent dosages (100 ms exposure; color bar: 4000 to 55,000; ∼30 min post‐injection). Scale bar: 1 cm. Reproduced with permission.^[^ ^111^ ^]^ Copyright 2017, Springer Nature. (C) images of lymphatic drainage about the hindlimb of nude mice using BTC1070. Reproduced with permission.^[^ ^129^ ^]^ Copyright 2019, Springer Nature. Scale bar, 2.5 mm. excitation: 1064 nm, emission: 1200–1700 nm. (D) High‐magnification (× 3) image of the ankle (red square in C), showing at least five dissociated collateral lymph vessels. Reproduced with permission.^[^ ^129^ ^]^ Copyright 2019, Springer Nature. (E, F) NIR‐IIb QDs enable high‐contrast lymph node imaging. Reproduced with permission.^[^ ^242^ ^]^ Copyright 2020, John Wiley & Sons.

It is crucial to detect accurately and identify the lymph nodes for the therapeutic and prognostic significance of tumors through direct imaging methods. Although large lymph nodes can currently be detected by cross‐sectional imaging modalities, non‐enlarged lymph nodes may also harbor metastases, and some enlarged lymph nodes may also be benign. NIR‐II fluorescence imaging possesses good promise for resolving resolution issues, while PET is still limited by the boundedness resolution.^[^ ^238^, ^239^ ^]^ Q8PNap/FBS has been successfully performed for imaging the lymphatic system of the hind limbs and small tumor metastases in mice.^[^ ^240^ ^]^ CH1055‐PEG has been used to image mouse inguinal lymph nodes in vivo, and the imaging performance was superior to that of ICG at the same tumor concentration.^[^ ^106^ ^]^ Nonetheless, the QY of CH1055‐PEG was only approximately 0.3%. To further improve the QY of CH1055‐PEG, the organic dye CH‐4T was designed to increase the fluorescence QY to 5%, which regulated the functional group of CH1055 from carboxylic acid to sulfonic acid. Furthermore, CH‐4T can combine with plasma proteins in human and bovine serum albumin (HAS and BSA) to form a CH‐4T complex (CH‐4T/HSA and CH‐4T/FBS) and the fluorescence intensity is 17‐fold higher than that of CH‐4T. Notably, when CH‐4T/FBS was heated at 70°C for 10 min (termed CH‐4T/FBS‐HT, QY ∼11%), the fluorescence brightness of CH‐4T/FBS‐HT was strongly enhanced to 28‐fold that of carbon nanotubes. Deep popliteal and sacral lymph nodes in mice have been achieved at the depth of 5–8 mm by using CH‐4T/HSA‐HT with NIR‐II imaging (Figure 11A,B).^[^ ^111^ ^]^ Most excitingly, IR‐12N3, as a NIR‐I dye in preclinical development, has been found to enhance the image resolution of lymphatic nodes with NIR‐II emission at 1200 nm, similar to the image resolution of CH‐4T/HSA‐HT.^[^ ^114^ ^]^ Meanwhile, the popliteal and sacral lymph nodes can be distinguished at a dorsal angle. Additionally, high‐resolution imaging of lymphatic drainage has been achieved by using small‐molecule NIR‐IIb fluorophore AIE HL3 dots.^[^ ^54^ ^]^ Another organic nanoprobe, IDSe‐IC2F NPs with NIR‐II fluorescence and photothermal properties provided PTT for metastatic lymph nodes.^[^ ^241^ ^]^ As an anti‐quenching cyanine fluorophore, CH1077 with low toxicity and body clearance mechanism exhibits stabilized absorption peak and emission peak in an aqueous solution. Furthermore, CH1077 has been used to image lymph nodes and could distinguish between the lymph nodes and the afferent and efferent lymphatic vessels (Figure 11C). Moreover, crowded collateral lymph vessels were also observed with a maximum SBR of 9.42 (Figure 11D). In addition, BTC1070 has excellent photostability, which facilitates the subsequent surgical removal of lymph nodes.^[^ ^129^ ^]^ Due to the spread of tumor cells via lymphatic vessels to far organs/tissues, sentinel lymph nodes (SLNs), as the first site of tumor cell metastases, play an important role in many tumors. If the SLNs do not contain any tumor cells, this indicates that the tumor has not spread and other lymph nodes do not need to be removed, and vice versa. Therefore, the rapid and accurate localization and identification of SLNs is a substantial challenge in tumor surgical treatment. Several methods have been developed for tracing lymph nodes. Two of the most widely used tracers are radioactive tracers and fluorophores (such as ICG, carbon nanoparticles, methylene blue, etc.). ICG is widely approved for NIR fluorescence imaging in clinical due to its lower level of autofluorescence and deeper tissue penetration capacity. Nevertheless, the ICG emission wavelength is endowed in the 700–900 nm range, which is significantly inferior to NIR‐II in vivo tracer imaging which has a wavelength range of 1000–1700 nm. Visualizing the process of tumor invasion into SLNs has been achieved by using bright NIR‐IIb fluorescence PbS/CdS core/shell QDs with dense polymer coatings. In addition, the QDs showed superior brightness and photostability in comparison to those of ICG (Figure 11E).^[^ ^242^ ^]^ Additionally, to facilitate the removal SLNs during surgery, oleyamine‐branched polyacrylic acid has been used to generate CD3@QDs probes‐by coating QDs with anti‐CD3 antibodies. These CD3@QDs specifically bind to T cells and extended the residence time of these cells in SLNs, enabling selective T cell targeting in the SLNs without further migration to adjacent lymph nodes (Figure 11F).^[^ ^242^ ^]^ Organic small‐molecule CQ‐T has been used to monitor real‐time lymphatic drainage and tumor angiogenesis. Moreover, the excellent optical properties of CQ‐T enable precise resection of tumors and SLN with NIR‐II navigation.^[^ ^243^ ^]^ Oral SCC has an intricate lymph node dynamic situation. Therefore, there remains challenging to identify metastatic lymph nodes during the operation. HT@CDDP NP has been used to effectively detect affected lymph nodes in a mouse model of orthotopic oral SCC, especially those that could otherwise have been easily overlooked.^[^ ^244^ ^]^ Agglomeration was easily observed in an aqueous solution by introducing a [2,2] paracyclophane group in the meso‐position of BODIPY. SLNs with a diameter of < 1 mm have been imaged and then removed precisely by utilizing the fluorescence signal from PCP‐BDP2 NPs.^[^ ^245^ ^]^ To date, though plentiful NIR‐II fluorophores have been performed for lymph node imaging,^[^ ^246^, ^247^ ^]^ fluorophores remain a biological safety problem. Hence, it is necessary to continue to develop rapidly excreted, nontoxic fluorophores.

### 4.2 NIR‐II imaging of distant tumor metastases

**FIGURE 12:**
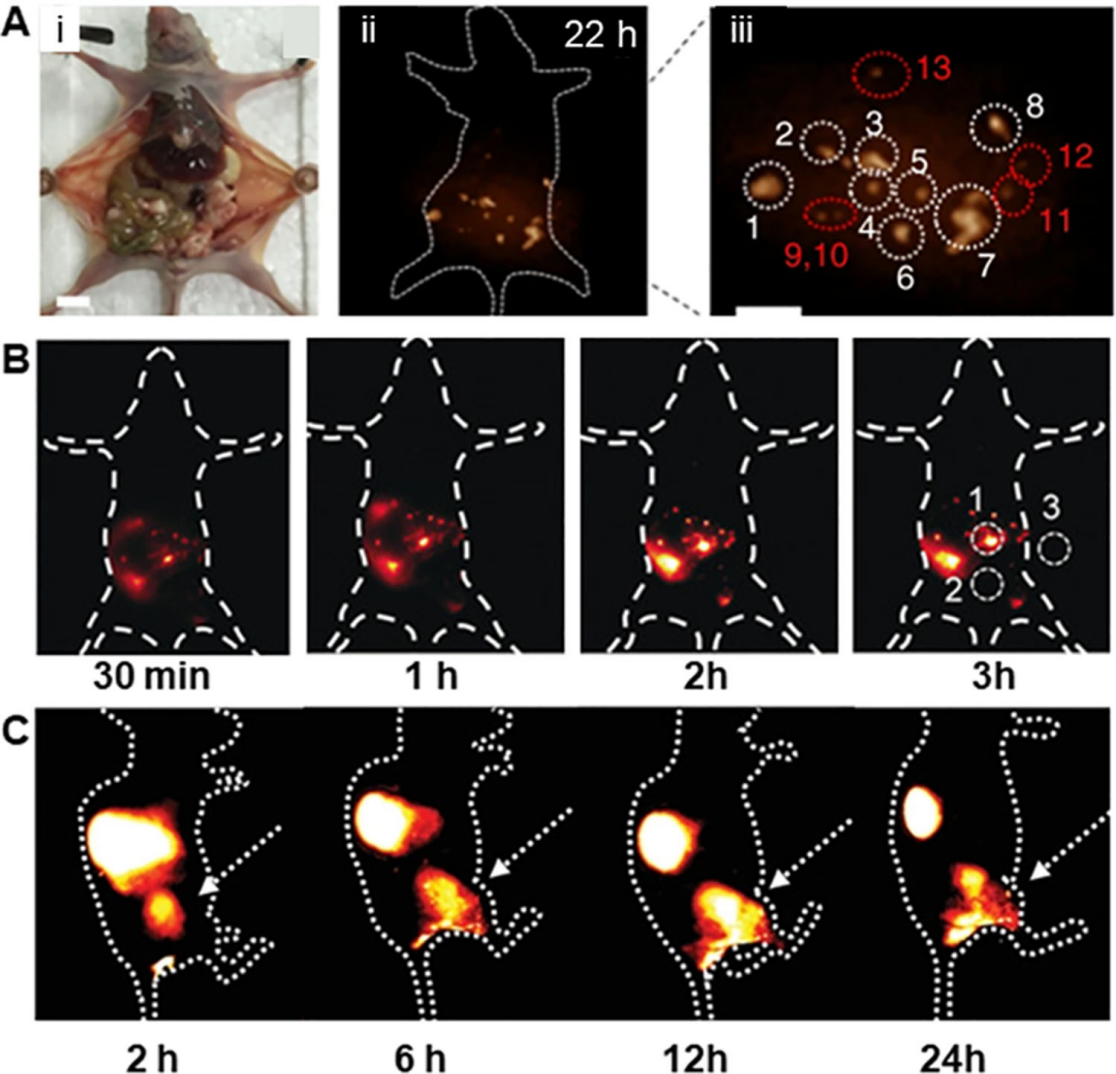
Imaging of distant tumor metastasis. (A) (i) NIR‐II imaging of human ovarian adenocarcinoma peritoneal metastases using DCNPs‐L_1_‐FSH_β_ nanoprobe. (ii) Imaging was obtained at 22 h; (iii) the enlargement of the NIR‐II nanoprobes with large peritoneal metastatic tumors (nos. 1–8) and ultra‐small lesions (nos. 9–3). 1000 nm LP filter; Scale bar, 1 cm. Reproduced with permission.^[^ ^180^ ^]^ Copyright 2018, Springer Nature. (B) The peritoneal metastasis tumor nodules were lighted by FEAD1. Reproduced with permission.^[^ ^255^ ^]^ Copyright 2020, John Wiley & Sons. (C) Lung metastasis from primary osteosarcoma was imaged using CH1055‐PEG. The orthotopic tumor has been indicated by white arrows. Reproduced with permission.^[^ ^257^ ^]^ Copyright 2020, John Wiley & Sons.

Systemic metastatic disease is the most widespread reason for death among tumor patients. Therefore, early detection of tumor cells/tissues can greatly improve the efficacy of cancer therapy. Dissemination of tumor cells from primary to distant sites often occurs at the very early stages of the disease.^[^ ^248^, ^249^ ^]^ At present, X‐ray, CT, MRI, US, and PET are commonly used for the clinical diagnosis of tumor metastasis. Nonetheless, these imaging strategies have been limited due to poor sensitivity and signal specificity, making it impossible to detect small metastases. In comparison, in vivo fluorescence imaging has been confirmed as a promising technique for improving the diagnosis of microscopic metastatic tumors, observing the response of tumors to therapy, and identifying recurrent or residual diseases.^[^ ^250^, ^251^ ^]^ NIR‐II fluorescence imaging in particular offers the possibility of finding metastases smaller than a few millimeters that are undetectable by current clinical imaging strategies.^[^ ^251^ ^]^ NIR‐II‐BPs have been used to visualize such metastases (1–2 mm) with high precision (T/N > 17.3).^[^ ^252^ ^]^ Ovarian cancer peritoneal metastases with < 1 mm have been detected with the DCNPs‐L_1_‐FSH_β_ nanoprobe, and the T/NT ratios of all metastatic tumors remained at ∼11 (Figure 12A).^[^ ^180^ ^]^ Gd‐REs@Lips possessed powerful functions in MRI and NIR‐II, allowing the visualization of small metastatic lesions (2 mm) on the liver surface.^[^ ^253^ ^]^ Due to the many tumor nodules buried in the deep tissues of peritoneal organs in peritoneal metastasis disease, it is crucial to distinguish tumor nodules and normal organs for successful surgery. APP‐Ag_2_S‐RGD has been used to detect nonvascularized small tumor peritoneal metastatic foci as small as approximately 0.2 mm in diameter.^[^ ^254^ ^]^ Furthermore, the nanoprobe FEAD1 has been used to diagnose and treat peritoneal metastases based on tumor microenvironment activation (Figure 12B).^[^ ^255^ ^]^ In addition to the peritoneum, the lung is also a common metastatic organ for tumors. The acceptor‐engineered squaraine dye SQ1 has been constructed with fibronectin and used to image breast cancer lung metastases.^[^ ^256^ ^]^ The metastatic nodules in the lungs from primary osteosarcoma have been visualized by applying CH1055‐PEG‐affibody in NIR‐II imaging (Figure 12C).^[^ ^257^ ^]^ Successive metastasis from primary breast tumors to the lymph nodes and then to the lungs has been dynamically imaged by using an NP‐Q‐NO_2_ nanoprobe which responds to nitroreductase and transforms into an activated D‐π‐A structure possessing a NIR‐II fluorescence.^[^ ^258^ ^]^ As AIEgens, BPBBT NPs have been used for the intraoperative localization of primary mouse colon tumors and metastatic parts. Thus, the intense photothermal conversion effect of BPBBT NPs was found to result in a thorough treatment of mice with colon cancer by utilizing the NIR‐II fluorescence imaging after optimized PTT.^[^ ^259^ ^]^

### 4.3 NIR‐II imaging surgery navigation

**FIGURE 13:**
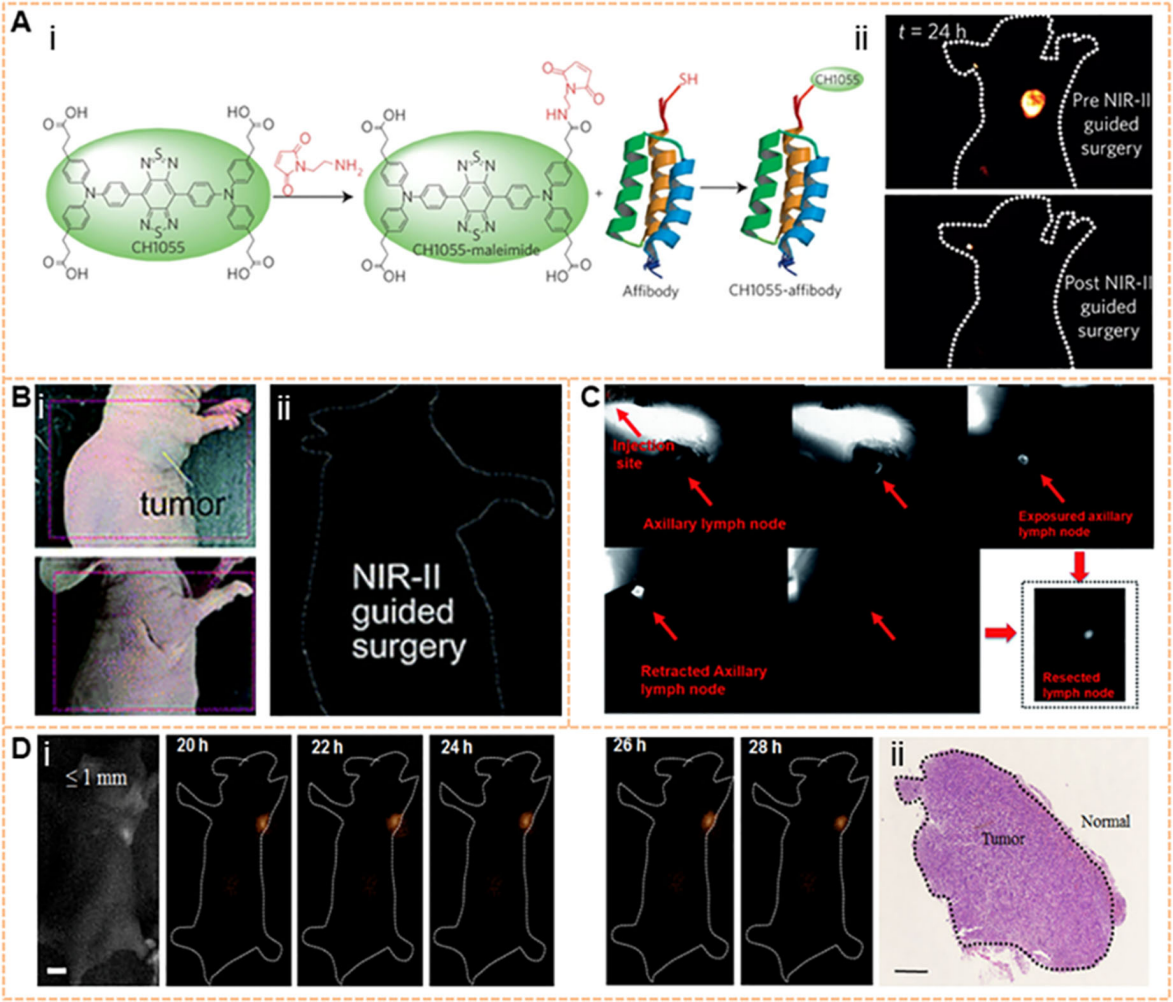
Tumor surgery navigation on NIR‐II window. (A) CH1055‐EGFR guided‐surgery NIR‐II fluorescence imaging setup and imaging‐guided tumor surgery. Reproduced with permission.^[^ ^106^ ^]^ Copyright 2016, Springer Nature. (B) H1 NPs for lymph node imaging and image‐guided surgery on the C57BL/6J model (1000 LP and 200 ms). Reproduced with permission.^[^ ^166^ ^]^ Copyright 2019, Royal Society of Chemistry. (C) NIR‐II imaging‐guided surgery to excise the tumor with IR‐BEMC6P@TATE. Reproduced with permission.^[^ ^108^ ^]^ Copyright 2017, Royal Society of Chemistry. (D) The resection was operated in the optimal surgical time window (Scale bar, 5 mm). The H&E staining result confirmed the tumor margin, and the tumor size was ≤1 mm (Scale bar, 0.1 mm). Reproduced with permission.^[^ ^180^ ^]^ Copyright 2018, Springer Nature.

Surgical resection of malignant tumors is one of the most widespread and efficient cures for tumors and is often the only treatment option.^[^ ^24^, ^260^ ^]^ Complete tumor resection has significant clinical value and incomplete tumor resection is usually associated with a high recurrence rate and poor prognosis.^[^ ^261^ ^]^ Accurately locating the tumor, subsequently detecting micrometastases, and completely resecting the tumors surgically are critical processes of successful tumor surgery. Researchers have also performed many studies on surgical navigation by using NIR‐II window imaging.^[^ ^262^, ^263^, ^264^, ^265^, ^266^ ^]^ Among them, CH1055, an organic SMD, was the most widely used NIR‐II dye because of its unique structure and good biosafety for targeted tumor imaging by binding protein/peptide segments.^[^ ^267^ ^]^ For example, CH1055 has been conjugated with anti‐EGFR (Figure 13A),^[^ ^106^ ^]^ urokinase plasminogen activator surface receptor,^[^ ^268^ ^]^ and Mn^2+^‐Apo‐Lf‐PEG.^[^ ^269^ ^]^ In addition, the benefit of NIR‐II fluorophores with superior T/NT ratios facilitated accurate surgical navigation of tumor removal.

First, NIR‐II fluorescence imaging shows promise for aiding tumor localization preoperatively.^[^ ^270^, ^271^ ^]^ Gd‐Ag_2_S and A1094@RGD‐HBc are unique dual‐modality nanoprobes that have been used to delineate brain tumors preoperatively.^[^ ^272^, ^273^ ^]^ Moreover, Gd‐Ag_2_S integrates the advantages of deeper penetration depth with the strong MR imaging of Gd.^[^ ^273^ ^]^ Another DSNPs@FSHP selectively exhibits efficient ovarian tumor‐targeting properties.^[^ ^274^ ^]^ The brightest organic dye, IR‐FEP, can identify xenograft 4T1 tumors with 100% accuracy. Furthermore, tumors have been precisely removed under surgical navigation by using NIR‐II fluorescence imaging.^[^ ^275^ ^]^ The nanocomposite probe BH‐NO_2_@BSA, which specifically responds to nitroreductase, can accurately locate orthotopic liver tumors preoperatively.^[^ ^276^ ^]^ XB1034‐cetuximab‐TCO was used for visible tumor delineation during NIR‐II imaging and PET imaging through a bioorthogonal reaction.^[^ ^277^ ^]^ IR‐BEMC6P@TATE specifically targeted the location of gastrointestinal neuroendocrine tumors and reduced the exposure of normal tissues. The margins of tumors could be clearly distinguished under NIR‐II imaging and white light. Moreover, IR‐BEMC6P@Tate was more accurate for tumor localization than PET (Figure 13B).^[^ ^166^ ^]^ Avoiding normal organ damage during surgery remains a huge challenge in clinical practice. Surgical success and safety will be significantly improved through multi‐channel fluorescence imaging in surgical platforms. Researchers have labeled LNs by combining NIR‐II fluorescent IDSe‐IC2F NPs possessing various optical properties. In addition, the surrounding typical structures, such as blood vessels and ureters, were visualized which avoids unnecessary injury to peripheral nerves, blood vessels, tendons, and other tissues.^[^ ^241^ ^]^ NIR‐II 2TT‐oC6B dots have also been performed to identify ureters to avoid intraoperative ureteral injury.^[^ ^57^ ^]^ Cancer cell membrane‐coated RE NPs (CC‐Nd@PEG) can emit strong 1060 nm fluorescence and achieve highly specific tumor imaging by labeling immune cells escape from the cancer cell membrane. In addition to targeting homologous cancer cells, CC‐Nd@PEG provides the surgeon with real‐time and precise feedback.^[^ ^75^ ^]^ PFTQ‐PEG‐GD NPs can locate the tumor site and treat tumors by using PTT, which can be visualized by integrating PA and T‐1‐weighted MRI.^[^ ^278^ ^]^ Cervical cancer has been excised under intraoperative NIR‐II fluorescence navigation by using a single AIEgen DDTB‐DP NP with a strong NIR fluorescence peak at 973 nm.^[^ ^279^ ^]^ As a bioconjugate NIR‐I dye, IR‐12N3 with bright NIR‐II tail emission increased the TNR from ∼4 to ∼10 in the NIR‐II range, exhibiting great prospects for improved tumor location.^[^ ^114^ ^]^

Second, NIR‐II fluorescence imaging has significant advantages for intraoperatively identifying tumor margins and evaluating the extent of tumor tissue removal. As ovarian cancer spreads mainly through the abdominal cavity, the complete removal of residual tumor tissues and obtaining minimal safety surgical margin are critical processes. With the application of DCNPS‐L‐FSHβ, peritoneal metastases of ovarian cancer can be accurately identified and wholly removed under NIR‐II image‐guided surgery.^[^ ^180^ ^]^ In addition, mice with ovarian cancer who were surgically removed by performing NIR‐II molecular imaging survived significantly longer than those whose tumors were removed by conventional surgery.^[^ ^280^ ^]^ The use of ZnGa_2_O_4_Cr_0.004_ (ZGC) nanoparticles during guided surgery can accurately delineate hepatocellular carcinoma.^[^ ^281^ ^]^ RBCP, a multipeak probe based on red blood cells, targets hepatocellular carcinoma for accurate tumor resection guided by NIR‐II imaging. Meanwhile, the release of laser‐activated O_2_ helped facilitate PDT for popliteal lymph node metastasis.^[^ ^282^ ^]^ As the emission tail of pNIR‐4 nanoparticles extends into the NIR‐IIa window, a pH‐responsive poly (β‐amino ester) facilitates the specific accumulation of pNIR‐4 at tumor sites, allowing NIR‐IIa fluorescence image‐guided precision resection of cancer.^[^ ^58^ ^]^ In C57BL/6J mice injected with H1 NPs, an SLN was faultlessly visualized with the help of determined NIR‐II imaging even when covered with soft tissue. In addition, the boundaries of the SLN could be handily recognized, eliminating unnecessary injury to the surrounding tissue (Figure 13C).^[^ ^108^ ^]^

An ideal imaging dye for tumor surgery navigation has the ability of rapid enrichment and low clearance in tumors to improve SBR and retention time, optimizing the surgical time window. Additionally, the dye should be quickly cleared from other organs of the body to reduce long‐term cytotoxicity. Zhang has conducted extensive research into the properties of imaging dyes.^[^ ^180^, ^283^ ^]^ DCNP@β‐CD was found to be retained and stable in tumors for as long as 5 h. In addition, liver retention was reduced 2.3 times compared with that of the assembly strategy by using 980 nm laser irradiation, yielding a higher bioimaging SBR of ∼15 and reducing biological toxicity.^[^ ^283^ ^]^ Moreover, the time interval between the first injection of DCNPs‐L_1_‐FSH_β_ and the second injection of complementary nanoprobe DCNPs‐L_2_‐FSH_β_ (second) was rationally designed for micro‐ovarian cancer (≤ 1 mm) resection. From 20 h to 26 h after the first injection, NIR‐II imaging and MRI detection showed good agreement, demonstrating a stable “optimal operative time window” of approximately 6 h for ovarian tumor resection (Figure 13D).^[^ ^180^ ^]^ NIR‐II bioimaging visualized the complete outline of tumors of various sizes in this superior time window. The optimal window for surgery has also been evaluated in other studies. PAA‐C/S nanoparticles with strong fluorescence emission at 1525 nm and high QY provided a 24 h surgical time window.^[^ ^160^ ^]^ Robust multifunctional fluorescent reagents play a vital role in successfully implementing multimodal image‐guided synergistic tumor treatment. One AIEgen, DDTB, has been designed for NIR‐II imaging, PTT, and PDT, producing immunological effects. Many tumors are removed by adequately using fluorescence navigation during surgery with this approach. Thereafter, PTT and PDT can eliminate many microscopic residual tumors to maximize the removal of tumor cells and tissues. Notably, the nanoparticle‐mediated PTT/PDT plus PD‐L1 antibody promoted tumor elimination by improving the function of immunotherapy.^[^ ^279^ ^]^

Tumor embolization surgery is a crucial method of tumor treatment. An embolization agent is injected into the target artery of the tumor blood supply through a catheter to occlude the target artery and block the tumor blood supply, causing tumor ischemia and necrosis. Eventually, tumor cells can be killed by infusing chemotherapy drugs. NIR‐II image‐guided tumor embolization surgery of femur orthotopic osteosarcoma has been performed in a nude mouse by injecting RENPs@Lips. Under the guidance of NIR‐II images, the central blood supply of the tumor was discriminated from the femoral artery. By simulating clinical vascular embolization which is the most common cause of death, blood flow was blocked by a vascular clamp until the blood flow signal of the tumor disappeared completely, which proved that the tumor blood supply was successfully blocked.^[^ ^284^ ^]^ These findings indicate the potential use of NIR‐II for tumor angioembolization navigation.

## 5 CONCLUSION AND OUTLOOK

**FIGURE 14:**
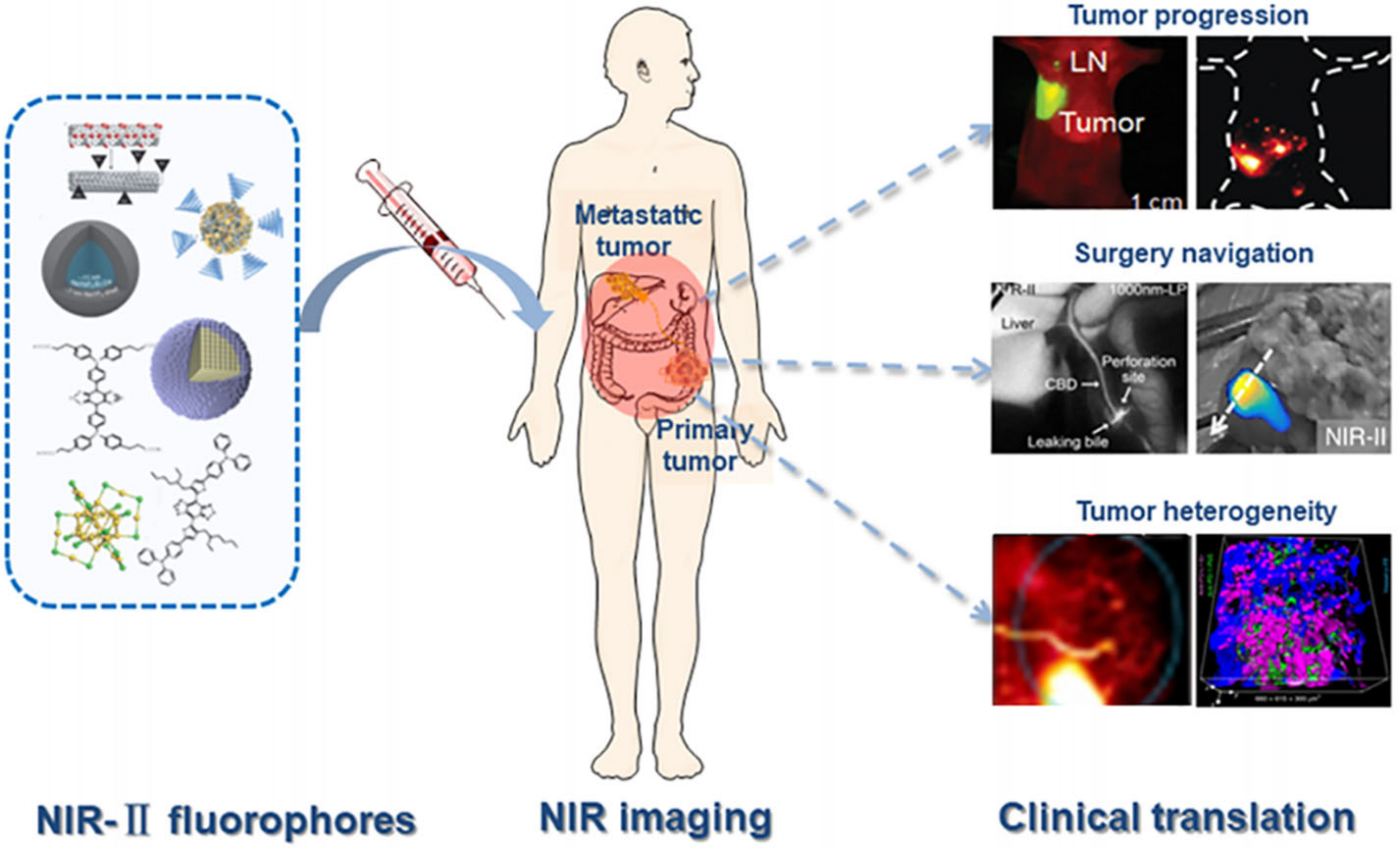
Overview of NIR‐II imaging for tumor. With the superior performance in terms of tissue penetration depth and spatiotemporal resolution, NIR‐II imaging has become a powerful imaging tool for in vivo detection. For oncology research, the heterogeneity in breast cancer has been detected by multi‐light path composite imaging, and the heterogeneity distribution of CD8^+^T cells in colon cancer has been imaged. At the same time, the imaging exploration of the microstructure in tumors provides the possibility for future research on the heterogeneity of tumors. In terms of tumor progression, it has shown a very high sensitivity to detecting lymphatic metastasis and distant metastasis of tumors. Reproduced with permission.^[^ ^196^, ^208^, ^242^, ^255^, ^293^, ^295^ ^]^ Copyright 2019, Ivyspring International publisher (tumor heterogeneity);Copyright 2019, Springer Nature (tumor heterogenity); Copyright 2020, John Wiley & Sons (tumor progression); Copyright 2020, Ivyspring International publisher (surgery navigation).

**FIGURE 15:**
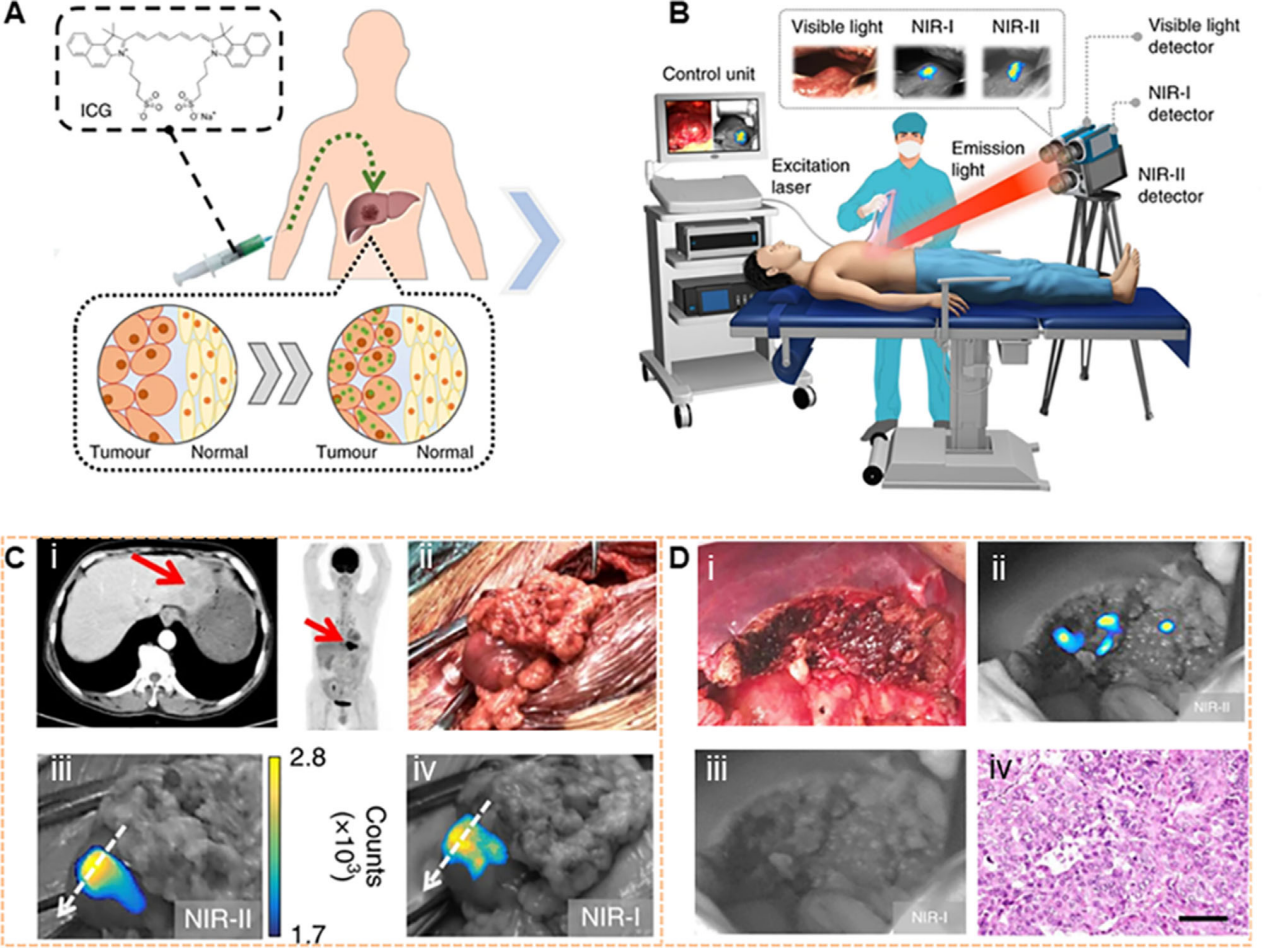
The clinical translation of NIR‐II imaging. Reproduced with permission.^[^ ^293^ ^]^ Copyright 2020, Springer Nature. (A) Before surgery, the patients were injected with ICG intravenously at a dose of 0.5 mg kg^−1^ body weight. (B) The integrated visual color‐NIR‐I NIR‐II imaging system. (C) Primary liver cancer in liver segments can be displayed by preoperative CT and PET images, and there was no extrahepatic metastasis. However, intraoperative NIR‐II and NIR‐I imaging found metastatic lesion in the omentum. (D) The tumor was thought to be removed entirely based on ultrasonography and the visible‐light image. H&E staining image: Scale bar, 50 μm.

Over the past 10 years, various NIR‐II probes have been developed for biological imaging at the macro scale, mesoscale, and micro scale.^[^ ^60^, ^110^, ^285^, ^286^, ^287^ ^]^ Recognized as an influential technique for tumor diagnosis and treatment in surgery, NIR‐II imaging has been propelled into a revolutionary period, which can be used in primary medical discussion and medical interventions by allowing the visualization of specific organs or tissues.^[^ ^288^, ^289^, ^290^, ^291^, ^292^ ^]^ As shown in Figure 14, different types of NIR‐II fluorophores have different advantages that can be taken advantage of to develop an essential function for realizing the heterogeneity and progression of tumors. Assisted by NIR‐II imaging probes and robot technology, surgeons can rely on visual cues provided by imaging devices rather than the naked eye to improve the efficiency of many surgical procedures. In 2018, a study of ICG for surgical navigation at the NIR‐II window was carried out.^[^ ^119^ ^]^ Recently, a surgery guided by the NIR‐II image system was performed in the clinic, which is the first time that NIR‐II imaging technology has been used for surgical navigation in the human body. The conventional NIR‐I dye ICG was observed to display tail fluorescence in the NIR‐II window. Then, these primary and metastatic liver tumors from 23 patients were removed by using ICG to aid fluorescence‐guided surgical resection.^[^ ^293^ ^]^ In this study, by comparing the clinical imaging performance of HCC patients under the NIR‐I window and NIR‐II window, it was found that NIR‐II imaging showed a clear contrast of tumor imaging and displayed the ability to detect primary liver tumors and metastases. These results demonstrated the tremendous clinical potential of NIR‐ΙΙ fluorescence imaging to guide surgeons during surgery (Figure 15).^[^ ^294^ ^]^ In addition, sufficient biliary structures can be visualized by using the fluorescence cholangiography at the NIR‐II window, which is an effective strategy to reduce the risk of cholecystectomy in terrible cases.^[^ ^295^ ^]^ However, as a non‐targeting fluorophore, ICG is not an optimal cancer tracer because it cannot distinguish between malignant and benign lesions. Additionally, ICG bound to serum proteins in the blood is mainly absorbed by the liver, which limits the development of ICG in surgery.

In addition to bioconjugation methods, other methods are being developed for the targeted imaging of tumors, such as dual pathological parameters synergistic,^[^ ^296^ ^]^ activatable NIR‐II fluorescence nanoprobes activated by using a “dual lock‐and‐key” for ultrahigh specific imaging of tumors in vivo. It is an emerging targeting strategy to achieve coating with cancer cell membrane components and utilize the functional homotypic tumor‐targeting capability from the source cell membrane for the NIR‐II fluorophore.^[^ ^297^ ^]^ The NIR‐II fluorophores with ignitability respond to the tumor microenvironment, improving the precision of tumor diagnosis.^[^ ^298^, ^299^, ^300^, ^301^, ^302^, ^303^ ^]^ For example, colorectal tumors could be accurately identified by using the NIR‐II@Si probe possessing deep‐tissue imaging of H_2_S‐rich colon cancer cells in animal models.^[^ ^304^ ^]^ A Mo_2_C‐derived polyoxometalate enabled specific tumor‐targeting imaging under the acidic tumor microenvironment.^[^ ^305^ ^]^ A lanthanide–cyanine Förster resonance energy transfer nanosensor, DSNP@MY‐1057‐GPC‐3, can respond to the tumor microenvironment (peroxynitrite, ONOO‐), showing the ability to detect in situ hepatocellular carcinoma accurately.^[^ ^306^ ^]^ Since tumors are heterogeneous diseases, energy metabolism in their microenvironment is constantly changing. The component ratio of NIR‐II emission aza‐BODIPY donors and pH‐sensitive rhodamine‐based pre‐acceptor in the Förster resonance energy transfer system can be regulated by pH transition adjustable sensors (pTAS), which can be used to dynamically monitor the pH in tumor lesions.^[^ ^307^ ^]^ Other approaches confirmed that the thiopyrylium cation can be utilized as a NIR acceptor to achieve the construction of novel mitochondria‐targeted NIR‐II chemotherapeutic and photothermal therapeutic dye H4‐PEG‐Glu.^[^ ^308^ ^]^ H4‐PEG‐PT could image mitochondria in osteosarcoma cells. Furthermore, the optical energy could be converted into heat energy to achieve mitochondria‐targeted PTT without reactive oxygen species effects.^[^ ^309^ ^]^

**FIGURE 16:**
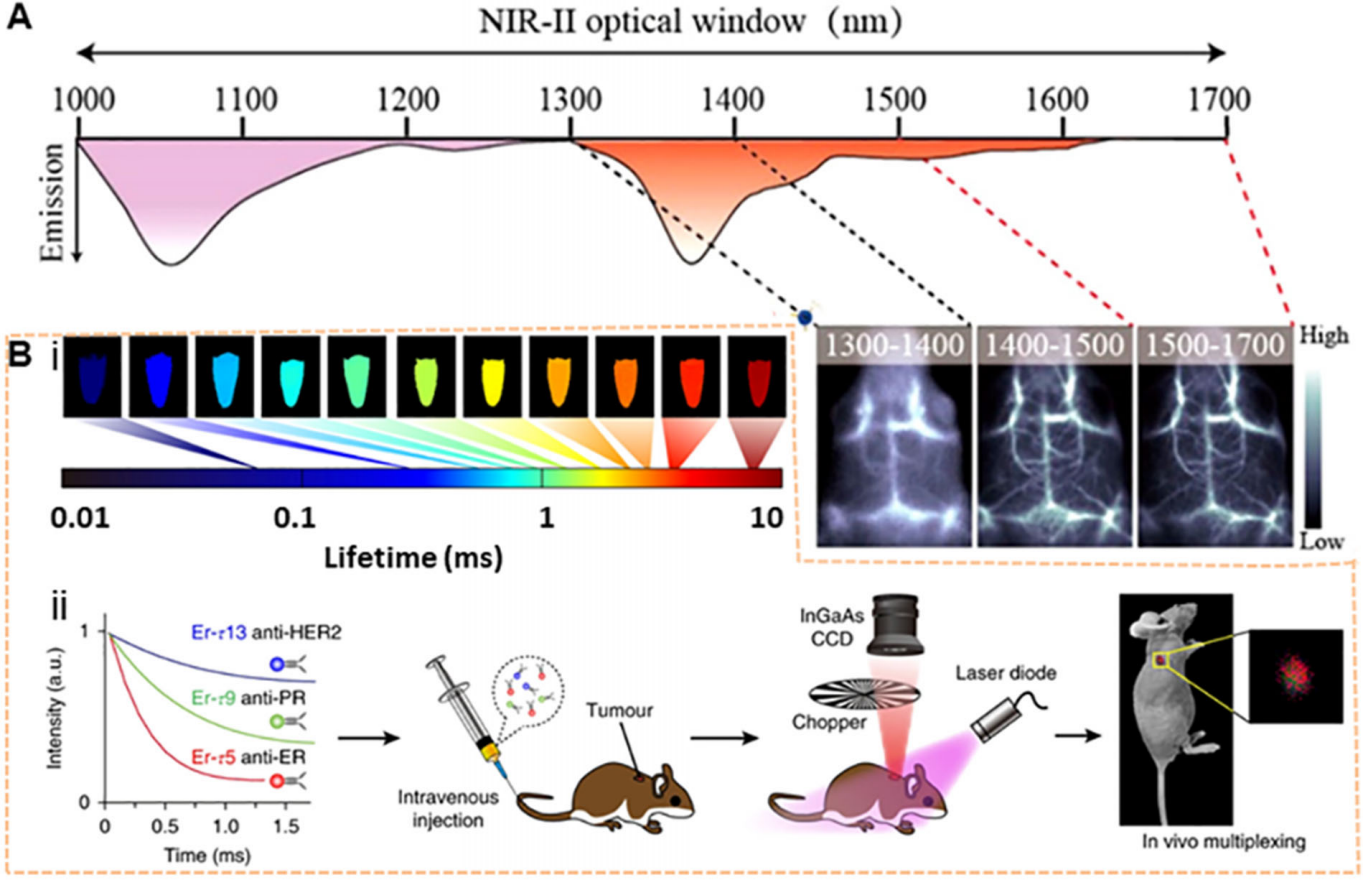
Outlook of the NIR‐II imaging. (A) The development of nanoparticles with good histocompatibility with wavelength over 1500 nm will significantly improve the resolution of in vivo optical, and biological imaging. Reproduced with permission.^[^ ^127^ ^]^ Copyright 2019, American Chemical Society. (B) Lanthanide‐doped NIR‐II nanoparticles, with an engineered luminescence lifetime, are used for heterogeneous breast cancer imaging by using time‐domain multiplexing quantitative imaging in vivo. Reproduced with permission.^[^ ^76^ ^]^ Copyright 2018, Springer Nature.

Although researchers are making efforts to develop ideal NIR‐II fluorescence agents, further optimization will be required for these probes to be applied in the clinic.^[^ ^310^ ^]^ These probes should possess high QY, significant absorption coefficient, good aqueous solubility, specific functional groups, and excellent pharmacokinetics. More importantly, biocompatibility is a key factor in the clinical translation of fluorophores. It is critical to develop fluorophores without in vivo toxicity by optimizing the structure, such as avoiding toxic chemical groups, using safety metal elements, enhancing the stability and solubility of the fluorophores, etc. For the nanostructures, controlling the hydrodynamic sizes below the cut‐off value of renal filtration (5.5 nm) is a feasible method to reduce the cumulative toxicity of the probes. In addition to the above aspects, tumor NIR‐II imaging has the following room for improvement:

1. Good compatibility of long‐wavelength (>1500 nm) organic small‐molecule probes. It is challenging to develop organic molecules by simply changing the structure to lengthen the absorption and emission wavelengths beyond the 1500 nm window. Among the currently available molecules, a new method for preparing NIR‐II J aggregates has been presented, which brings great inspiration for other NIR‐II molecular dyes, achieving excellent biological imaging at longer wavelengths (Figure 16A).^[^ ^127^ ^]^
2. NI>R‐II real‐time multichannel imaging. To date, in vivo NIR‐II imaging usually used a single probe, or occasionally two or three, providing a preliminary assessment of the quantitative accuracy of the probes.^[^ ^46^, ^186^ ^]^ Multiplex probes based on unique lifetime channels, such as engineered NIR‐II lanthanide fluorescence probes, have been developed to enable in vivo multiple imaging and accurate detection of breast cancer heterogeneity (Figure 16B).^[^ ^76^ ^]^ In the future, more methods will be developed for real‐time multichannel imaging.
3. Optimizing the binding moiety of NIR‐II probes. Based on bioorthogonal chemistry, NIR‐II tumor‐targeted molecular imaging can be conducted. To satisfy the demands of biorthogonality, different chemical linking technologies have been investigated. The definition of bioorthogonal chemistry can be introduced into molecular imaging by modifying NIR‐II fluorophores with reactive functional groups (such as cyclooctane, ketone, and tetrazine), but without altering their original optical properties as much as possible.
4. NIR‐II high‐precision microscopic imaging. For tumor heterogeneity imaging, targeted imaging has been carried out on tumors from different sources to distinguish the heterogeneity between tumors. The heterogeneity of histological phenotypes within the tumor has not yet been analyzed. Furthermore, the heterogeneity of molecular phenotypic changes in primary and metastatic tumors in the same patient has also yet to be explored. Multispectral long‐wavelength imaging methods combining multiple NIR‐II probes could provide an excellent strategy for comprehensively analyzing tumorigenesis, progression, and heterogeneity. At the same time, persistent luminescence may be directly applied in living tissues to monitor pathological processes.^[^ ^311^ ^]^ With the development of imaging instruments, the use of NIR‐II probes to achieve high‐precision imaging of tumor organelles will provide more sophisticated insights into tumor mechanisms.

## CONFLICT OF INTEREST STATEMENT

The authors declare no conflicts of interest.
